# Prevalence and Risk Factors for Spasticity After Stroke: A Systematic Review and Meta-Analysis

**DOI:** 10.3389/fneur.2020.616097

**Published:** 2021-01-20

**Authors:** Huangling Zeng, Jian Chen, Yang Guo, Sheng Tan

**Affiliations:** Department of Neurology, Zhujiang Hospital, Southern Medical University, Guangzhou, China

**Keywords:** prevalence, risk factors, stroke, spasm, meta-analysis

## Abstract

**Background:** Spasticity is a common sequela of stroke. The incidence of poststroke spasticity (PSS) has not been systematically reviewed in recent years, and some risk factors remain debated. This systematic review and meta-analysis was conducted to determine the prevalence and risk factors for PSS.

**Methods:** We searched electronic databases (PubMed, Embase, Cochrane Library, CNKI, WANFANG and CBM) inception to May 12, 2020. Observational studies summarizing the incidence or risk factors for PSS were included. Only cohort studies were enrolled in meta-analysis. For risk factors examined in at least three different studies, we combined effects into odds ratios (OR) and 95% confidence intervals (CI).

**Results:** One thousand four hundred sixty-seven studies were retrieved and 23 were involved in meta-analysis. The pooled prevalence of spasticity after stroke was 25.3% and that after the first-ever stroke was 26.7%. The incidence of spasticity after the first-ever stroke with paresis was 39.5%. The prevalence of disabling or severe spasticity (MAS ≥ 3) in stroke patients with paresis was 9.4% (95% CI 0.056–0.133), and severe spasticity was 10.3% (95% CI 0.058–0.149). Moderate to severe paresis (OR = 6.573, 95% CI 2.579–16.755, *I*^2^ = 0.0%), hemorrhagic stroke (OR = 1.879, 95% CI 1.418–2.490, *I*^2^ = 27.3%) and sensory disorder were risk factors for PSS.

**Conclusions:** The incidence of PSS was significantly higher in stroke patients with paresis. Patients with moderate to severe paresis and sensory disorder should be closely followed up. The role of hemorrhagic stroke in predicting PSS remains to be further explored.

## Introduction

Stroke is the third leading cause of death in the world, with about 795,000 people experiencing a new or recurrent stroke each year (1, 2). Spasticity is a common sequela of stroke patients with an incidence of 4–42.6% (3). Initially Lance defined spasticity as an increased velocity-dependent muscle tone with exaggerated tendon jerks caused by hyperexcitability of the stretch reflex (4). Pandyan limited spasticity to all the positive signs of the Upper Motor Neuron (UMN) syndrome and redefined spasticity as disordered sensorimotor control, presenting as intermittent, or sustained involuntary involvement of muscles (5). Recently Dressler proposed a new definition as involuntary muscle overactivity in central paresis, which is caused by slow or rapid passive joint movement or sensory stimulation (6). Poststroke spasticity (PSS) severely impairs upper-limb flexibility and the ability of walking and moving, mostly resulting from five characteristic arm spasticity patterns, four common ankle and foot spasticity patterns and stiff-knee gait (7–11). Long-term PSS may cause a severe deterioration of quality life due to complications including joint contractures, decubitus and pain (12, 13), leading to a fourfold increase in care burden (14).

A 2013 review reported that the incidence of PSS in early stroke was 4–27%, in post-acute stroke was 19–26.7%, and in chronic stroke was 17–42.6% (3). In recent years, the prevalence of PSS has not been aggregated or updated. And it may change with the improvement of health care and economy, resulting in calling for further research (15). Meanwhile, no literature has quantified summarized the prevalence of PSS. Data on the incidence of disabling spasticity with a need for interventions are still scarce. There is no generally accepted definition of disabling spasticity. Lundström defined disabling spasticity as spasticity that have a clinically significant impact on movement function, activity performance, or participation in social life, accompanied by positive symptoms of UMN syndrome (16). An international panel of clinical experts recommended building on clinical expertise and the concepts of the International Classification of Functioning, Disability and Health (ICF) domains (17), defined spasticity that considered by a spinal cord damage patients or caregiver to interfere with body function, activities and/or participation as disabling spasticity (18). This is consistent with another study (17, 19).

Patients with PSS who have low motor recovery potential may benefit from aggressive treatment and care (20, 21). Aiming at correctly following-up high-risk patients and effectively guide resources, a more comprehensive assessment of risk factors for PSS is needed (22). Severe paresis has been recognized as a risk factor for PSS in previous studies, but whether sensory impairment, early increased muscle tone, low BI score, stroke-related pain, hemorrhagic stroke and cognitive impairment predict PSS remains debated. A previous meta-analysis found motor and sensory impairment, hemorrhagic stroke and age predicted upper limb spasticity 1 month after stroke (23). But another review qualitatively concluded that low Barthel index (BI) score, severe paralysis, stroke related pain and sensory disorders were key risk factors for PSS (3). The differences may be related to inconsistent assessment methods and subjects.

Therefore, aiming at summarizing the latest evidences, this study provided a quantitative and qualitative analysis of the incidence of PSS including disabling spasticity and severe spasticity over time. To enhance evidence for the prevention and treatment of PSS, this meta-analysis was conducted to investigate risk factors for PSS. And a descriptive summary of risk factors that did not meet the inclusion criteria for meta-analysis was presented. Finally, the onset and course of PSS and the mechanisms involved in predicting PSS are discussed in this review.

## Materials and Methods

This meta-analysis was based on the guidelines for the preferred reporting project of systematic review and meta-analysis (PRISMA). We registered a protocol in PROSPERO under number CRD42020200948.

### Search Strategy

Publications were searched PubMed, Embase, Cochrane Library, CNKI, WANFANG and CBM up to May 12, 2020 and were supplemented by manually selected from reference list and review. The term words were included “stroke,” “muscle spasticity,” “spasticity,” “muscle hypertonia,” “dystonia,” “muscle tonus,” “epidemiology,” “prevalence,” “incidence,” “predict,” “determinant,” “morbidity,” “risk,” and “occurrence.” The detailed search strategy is presented in Supplementary Material. Only literature in English and Chinese were searched. Two authors separately and independently screened studies according to the selection criteria. A third author made the ultimate decision if consensus could not be reached. For studies without sufficient information to evaluate the eligibility, we contacted authors via E-mail to obtain data.

### Inclusion and Exclusion Criteria

Studies were included if met the following criteria: (1) Participants over 18 years old were had a stroke with a diagnosis by WHO criteria, excluding subarachnoid hemorrhage. (2) Outcomes included the risk factors and/or the prevalence of PSS. (3) Only cohort studies were enrolled in meta-analysis. Risk factors investigated in at least three studies that assessed muscle tone by the Modified Ashworth Scale (MAS) or the Ashworth Scale (AS) were meta-analyzed. Studies included in meta-analysis of the incidence of PSS were not required to use specific methods to assess spasticity. If multiple publications report the same results from the same study, the publication with the largest sample size was chosen. Conference literatures and studies focusing on functional improvement after intervention were excluded.

### Data Extraction

The following data were independently extracted and cross-checked by the two evaluators according to the predetermined table: study characteristics (publication year, first author, study design, country, sample size), subjects (stroke type), outcomes (spasticity events, risk factors), follow-up time. Based on the concepts of the ICF domains, we defined disabling spasticity as spasticity that have a clinically significant impact on movement function, activity performance, or participation in social life, accompanied by positive symptoms of UMN syndrome. Severe spasticity was defined as MAS greater than or equal to 3.

### Risk of Bias Assessment

The Newcastle-Ottawa scale (NOS) was used to assess the risk of bias in cohort studies through 8 items in three blocks, including selection, comparability, exposure evaluation, or outcome evaluation. NOS adopted the semi-quantitative principle of a star system in the quality assessment of cohort studies, with a full score of 9 stars. Cohort study was scored with six or more stars indicating a low risk of bias and less than 6 stars indicating a high risk of bias.

### Data Analysis

STATA 14.0 statistical software (StataCorp LP, College Station, Texas, USA) was used for statistical analysis. The pooled incidence of spasticity was calculated as a percentage based on the prevalence and standard error of each study, combining with a 95% confidence interval (CI). Risk factors were calculated by odds ratio (OR) and 95% CI. Bilateral *P* < 0.05 were considered statistically significant. Heterogeneity was measured by Cochran's Q test combined with *I*^2^ statistics. The level of heterogeneity represented by the *I*^2^ statistics is interpreted as small (*I*^2^ ≤ 25%), medium (25% < *I*^2^ ≤ 50%), large (50% < *I*^2^ ≤ 75%), or very large (*I*^2^ > 75%). A fixed-effect model was used if acceptable heterogeneity is found (*I*^2^ < 50%, *P* > 0.1). And random effect model was used to adjust significant heterogeneity. Subgroup analysis was performed according to different follow-up times including four periods: within 1, 1–3, 3–6, beyond 6 months. Sensitivity analysis excluded individual studies and re-conducted meta-analysis to evaluate the robustness of comprehensive results.

## Results

### Study Selection

**Figure 1:**
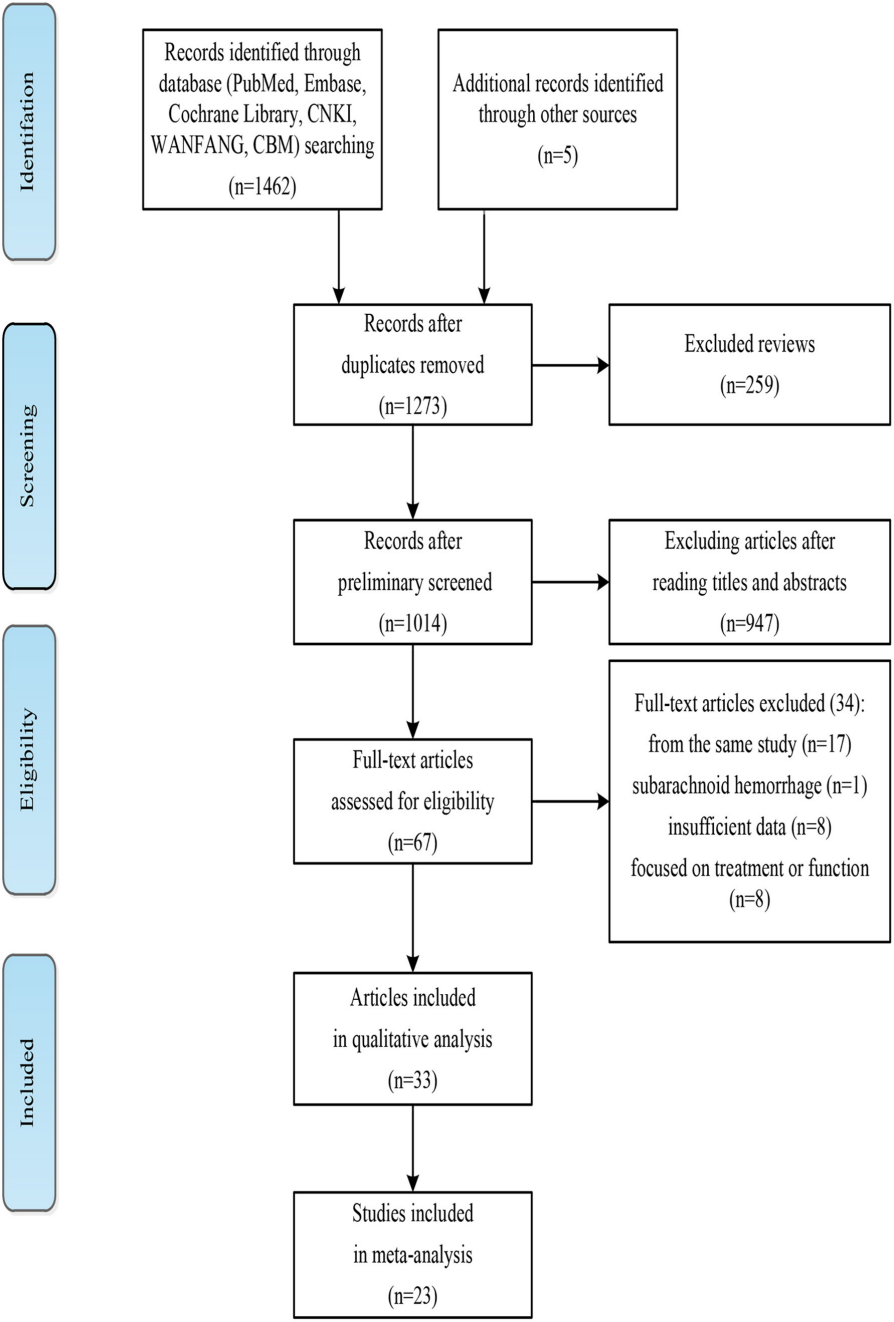
The literature search and study selection process.

Figure 1 shows the detailed literature search and filtering process. 1,462 articles retrieved and 5 studies were supplemented from review or reference lists. One hundred ninety four duplicate articles and 259 reviews were eliminated. After reading abstracts, 947 articles were excluded and 67 full-text articles were identified. Thirty four full-text articles were excluded for the following reasons: After contacting the authors, we were unable to obtain sufficient data from the nine studies; one included patients with subarachnoid hemorrhage; 17 were the same studies; 8 studies focused on treatment or predictive effect of PSS on function. Finally, 32 articles were analyzed qualitatively (16, 24–54). Twenty two cohort studies reported the incidence or risk factors for PSS were included in meta-analysis (24, 28–35, 41–43, 45, 46, 48–54). Walkins et al. and Lealthy et al. (38, 52), Opheim et al. (41, 42) were the same studies, but focused on the incidence or risk factors for PSS, respectively.

### Study Characteristics and Risk of Bias

Table 1 summarizes the characteristics of included studies in meta-analysis. Six thousand nine hundred and fifty six subjects were enrolled, and sample size ranged from 43 to 3056. Subjects were concentrated in Europe and Asia, with no participants from Oceania or Africa. Eleven studies included stroke patients, and nine studies included only ischemic stroke patients. Follow-up time ranged from 3 months to 7 years, with the majority in 3–6 months. The risk of bias was low in 21 studies with 6 stars or more. Only one study scored below 6, indicating a high risk of bias.

**Table 1:** Characteristics of included studies in meta-analysis.

**Study**	**Study design**	**Country**	**Sample**	**Stroke**	**Spasticity definition**	**Spasticity events**	**Predictors**	**Follow-up**	**Quality**
Volny et al. (51)	Prospective cohort study	Czech	76	First ever ischemic stroke	MAS > 1	44	Age, gender, baseline NIHSS, ASPECTS, location	6 months	6
Shin et al. (49)	Prospective cohort study	Korea	3056	First ever stroke	MAS ≥ 1	3, 6, 12 months: 156, 153, 160	Age, gender, stroke type,	12 months	7
Katoozian et al. (34)	Prospective cohort study	Iran	149	First ever stroke with hemiplegia	MAS ≥ 1	1 week: 26 1 month: 30; 3 months: 18	Age, gender, stroke type, sensory disorder, lesion site	3 months	6
Opheim et al. (41, 42)	Prospective cohort study	Sweden	117	First ever stroke with arm paresis	MAS ≥ 1	4 weeks: 48 12 months: 35	Age, sensation, paresis, early increase in muscle tone, OCSP, FMA	12 months	8
Kong et al. (36)	Prospective cohort study	Singapore	163	First-ever ischemic stroke with weakness	MAS ≥ 1 Severe PSS:MAS ≥ 3	3, 6, 12months: 54, 67, 72	UEMI, MBI	12 months	5
Lundström et al. (30)	Prospective cohort study	Sweden	50	First-ever stroke with paresis	MAS ≥ 1 Disabling spasticity	1 month: 13 6 months: 6	Severe arm paresis, sensory disorder	6 months	7
Persson et al. (43)	Prospective cohort study	Sweden	288	First-ever or recurrent acute ischemic stroke	MAS ≥ 2	99	Age, arm motor power	7 years	7
Li et al. (54)	Prospective cohort study	China	185	ischemic stroke	MAS ≥ 1 Severe PSS:MAS ≥ 3	32	Age, gender, sensory, smoking, diabetes, BI, lesion site, hypertension	6 months	6
Urban et al. (46)	Prospective cohort study	Germany	211	First-ever ischemic stroke with paresis	MAS > 1 Severe PSS:MAS ≥ 3	90	Severe paresis and hemi-hypesthesia at stroke onset, moderate paresis, mild paresis	6 months	9
Leathley et al. (38) Watkins et al. (52)	Cohort study	UK	106	Stroke	MAS ≥ 1	38	Leg and arm weakness, BI, smoking, side of weakness	12 months	7
Ryu et al. (48)	Retrospective cohort study	Korea	245	stroke	MAS ≥ 1 Severe PSS:MAS ≥ 3	104	NIHSS, Motricity index	No reported	5
de Jong et al. (28)	Prospective cohort Study	Netherlands	50	Ischemic stroke and paralysis	MAS ≥ 1+	3 months: 23 6 months: 26	FMA, stroke duration	6 months	6
Dornák et al. (29)	Prospective cohort study	Czech	307	First stroke with motor deficit	MAS > 1 Severe PSS:MAS ≥ 3	138	BI, mRS	12 months	8
van Kuijk et al. (50)	Cohort study	Netherlands	43	Ischemic stroke with paralysis	AS ≥ 2	22	Sensory disorder, early hand motor recovery	26 weeks	7
Plantin et al. (45)	Cohort study	Sweden	61	First ever stroke with weakness	Neuro Flexor	3 weeks: 20; 3, 6 months: 28	corticospinal tract damage, FMA-hand, lesion Volume	6 months	6
Jin et al. (33)	Cohort study	China	407	First ever ischemic stroke	MAS > 1	3, 6, 12 months: 142, 163, 173	Stroke site	12 months	6
Picelli et al. (24)	Retrospective cohort study	Italy	72	Ischemic stroke hemiparesis	Severe PSS:MAS ≥ 3	40	Age, gender, ESS foot, ESS arm (proximal paresis of upper limb and distal paresis of lower limb), smoking	6 months	7
Egen-Lappe et al. (31)	Cohort study	Germany	1167	Stroke	Diagnosis code	119	Age, gender, hypertension, diabetes, stroke type	6 months	6
Wissel et al. (32)	Prospective cohort study	Germany	94	Stroke	MAS ≥ 1	6 days: 23; 6, 16 weeks: 23,18	Paresis, lower BI score, early increase in muscle tone	16 weeks	6
Welmer et al. (53)	Prospective cohort study	Sweden	109	First-ever stroke	MAS > 0	3 months: 18 18 months: 13	No reported	18 months	6

*ASPECTS, the Alberta Stroke Program Early CT Score; BI, Barthel Index; ESS, European Stroke Scale; NIHSS, National Institute of Health stroke scale; MBI, Modified Barthel Index; FMA, the Fugl-Meyer Assessment; mRS, modified Rankin scale; FMA-UE, the Fugl-Meyer Assessment Upper Extremity Scale; MAS, the Modified Ashworth Scale; AS, the Ashworth Scale; PSS, Poststroke spasticity; UEMI, Upper Extremity Motricity Index; OCSP, Oxfordshire community stroke project*.

### Incidence of Spasticity

This review descriptively summarized the incidence of PSS in 24 cross-sectional and cohort studies in order to compare with previous reviews (3, 16, 24, 25, 27–36, 41, 45, 46, 48–54). The prevalence of PSS ranged from 4 to 46% within 1 month, 4.16–48% in 1–3 months, 6.9–63% in 3–6 months, 7.6–49% beyond 6 months. And 2–2.6% of patients developed disabling or severe spasticity within 1 month, 5% in 1–3 months, 8–15.6% in 6 months and 12.5–18% beyond 6 months. Since most previous studies considered paresis to be a risk factor for PSS, we divided studies into two groups based on inclusion criteria for concurrent paresis: general stroke is defined as the diagnosis of stroke, whether or not paresis occurs. It is addressed to the entire stroke population. Stroke with paresis was defined as a stroke accompanied by a stroke-induced paresis.

### Incidence of Spasticity in General Stroke

**Figure 2:**
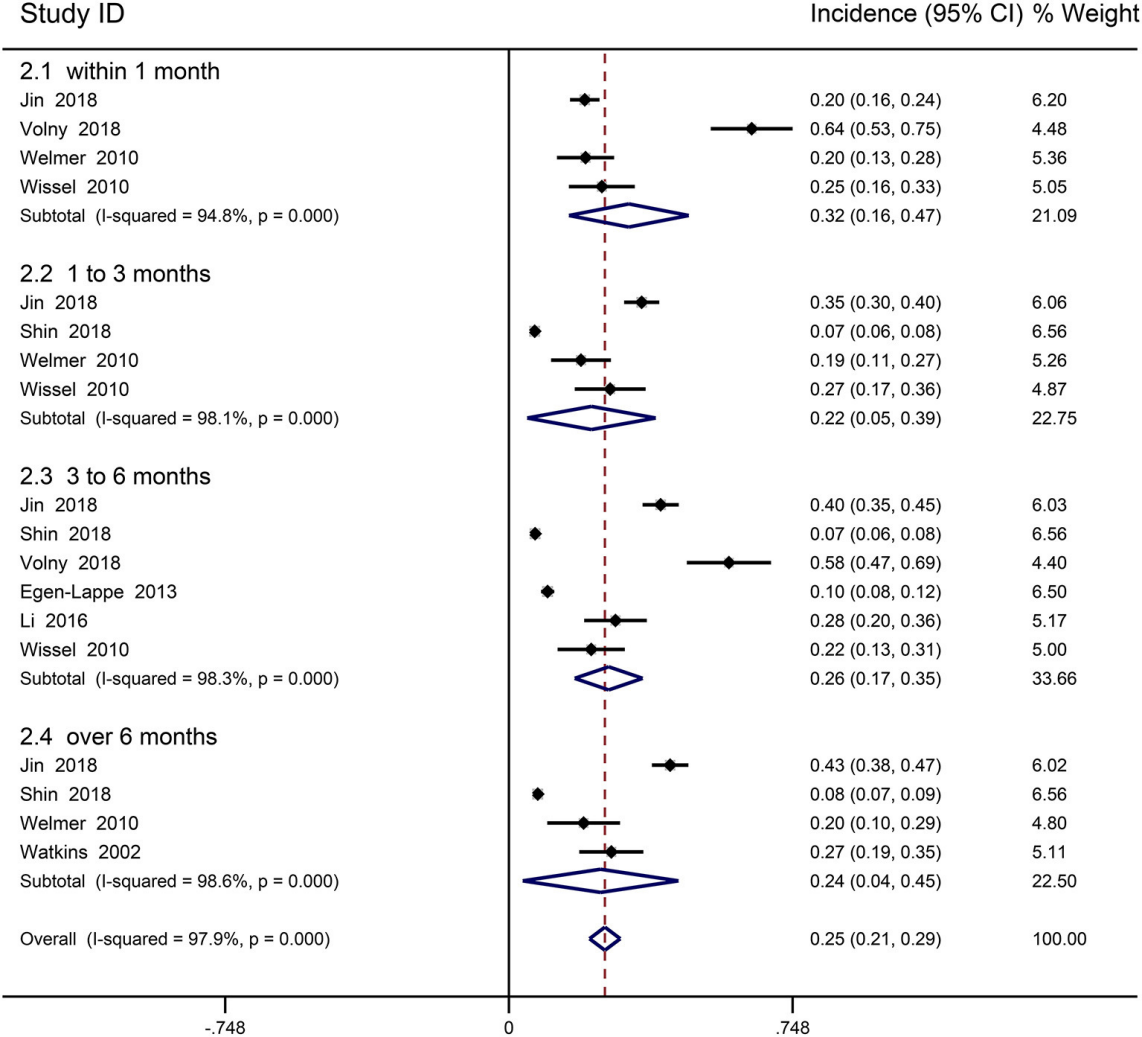
Forest plot for the prevalence of spasticity in general stoke patients.

Nine cohort studies reported the prevalence of spasticity in general stroke patients and were included in meta-analysis (31–33, 43, 49, 51–54). Ryu was excluded due to uncertain follow-up time (48). The prevalence of PSS was 6.8–64%. The follow-up period ranged from 6 days to 7 years, mostly within 12 months. Meta-analysis found that the incidence of PSS was the highest within 1 month, and the incidence changed little after 3 months. The overall incidence was 25.3% (95% CI 0.213–0.293, *I*^2^ = 97.9%; Figure 2), 31.6% (95% CI 0.158–0.474, *I*^2^ = 94.8%) within 1 month, 21.8% (95% CI 0.048–0.387, *I*^2^ = 98.1%) in 1 to 3 months, 26.3% (95% CI 0.174–0.353, *I*^2^ = 98.3%) in 3–6 months, 24.2% (95% CI 0.036–0.447, *I*^2^ = 98.6%) beyond 6 months. Five studies included patients with a first-ever stroke, and four did not identify patients with a first or recurrent stroke. Combined results showed that overall incidence of PSS in the first-ever stroke patients was 26.7% (95% CI 0.217–0.318, *I*^2^ = 98.5%). Most studies reported mild-to-moderate increases in muscle tone. Only two studies reported severe spasticity, with incidence ranging from 2 to 2.6% (48, 54).

### Incidence of Spasticity in Stroke With Paresis

**Figure 3:**
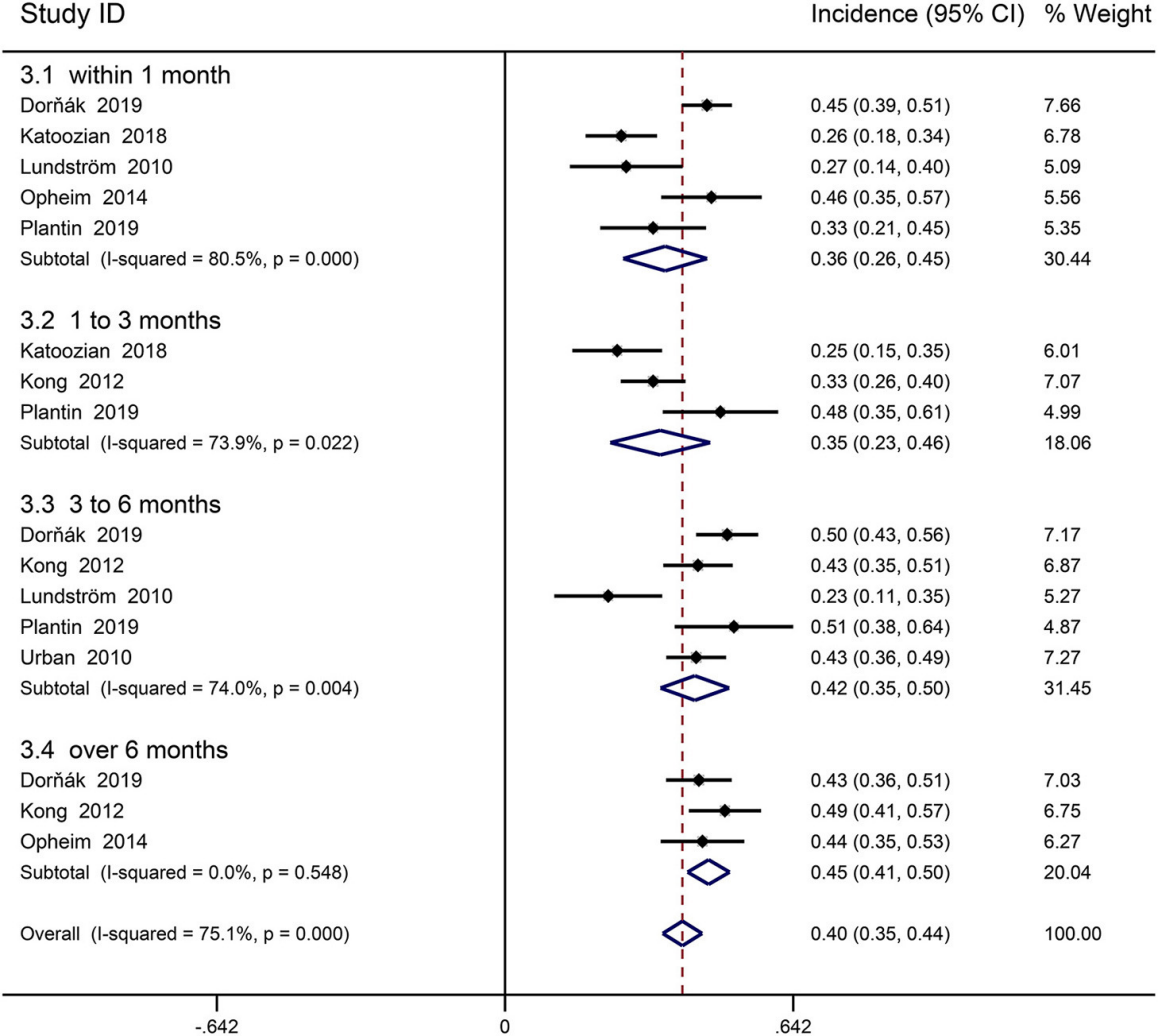
Forest plot for the prevalence of spasticity in first-ever stoke patients with paresis.

**Figure 4:**
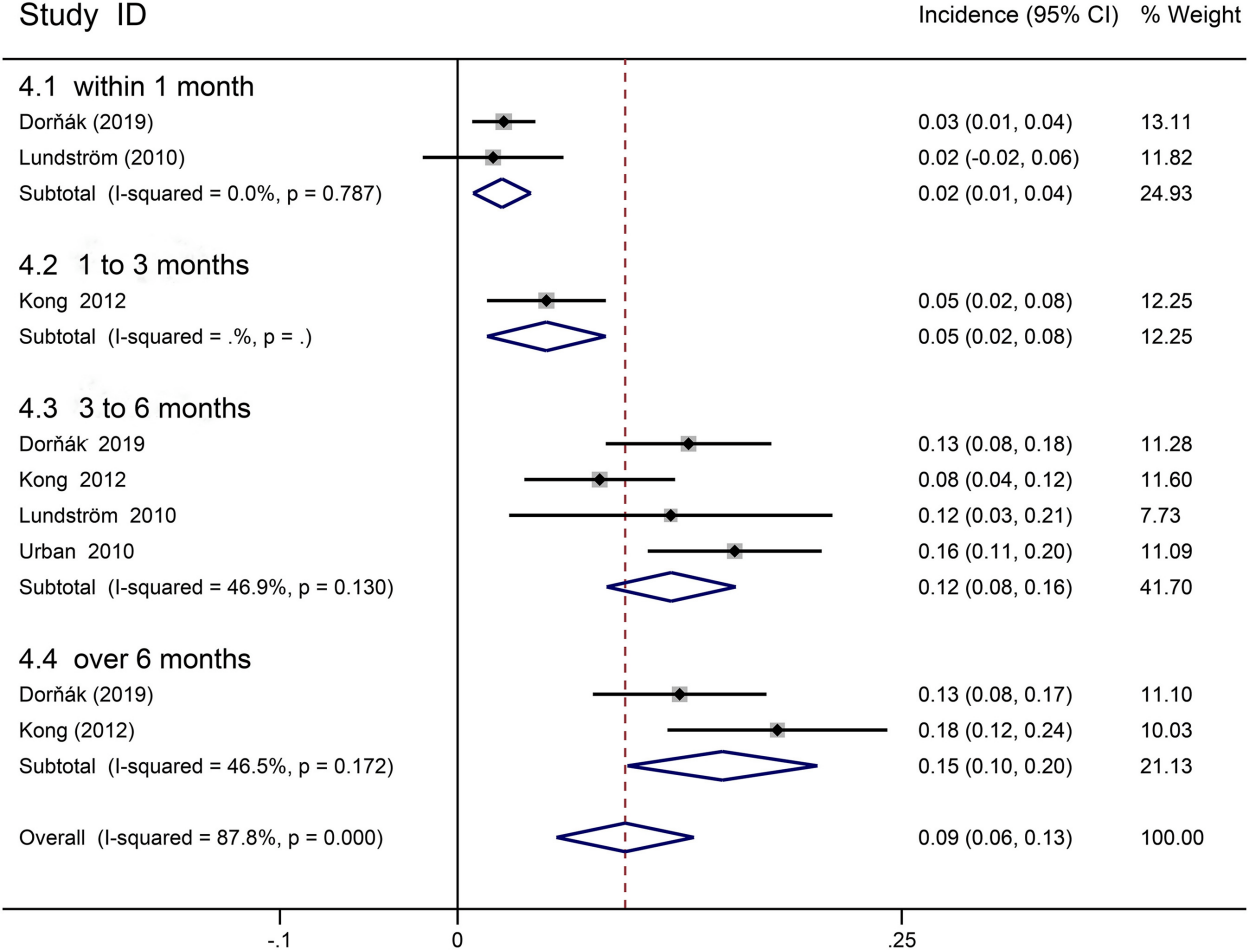
Forest plot for the prevalence of disabling or severe spasticity in stoke patients with paresis.

Seven cohort studies involving first-ever stroke patients with paresis provided data on incidence of PSS (29, 30, 34, 35, 41, 45, 46). Picelli was excluded from the meta-analysis because of the great heterogeneity, resulting from including stroke patients with early PSS in the rehabilitation center (24). The follow-up period ranged from 3 days to 12 months. Declarative review summarized that 23–49% of stroke patients with paresis had spasticity. Meta-analysis revealed an overall incidence of spasticity in first-ever stroke patients with paresis was 39.5% (95% CI 0.351 to 0.439, *I*^2^ = 75.1%; Figure 3), 35.7% (95% CI 0.263–0.450, *I*^2^ = 80.5%) within 1 month, 34.6% (95% CI 0.235–0.457, *I*^2^ = 73.9%) in 1–3 months, 42.3% (95% CI 0.346–0.500, *I*^2^ = 74.0%) from 3 to 6 months, 45.4% (95% CI 0.407–0.500, *I*^2^ = 0%) beyond 6 months. Three studies assessed the incidence of severe spasticity in stroke patients with paresis, and one reported the incidence of disabling spasticity (29, 30, 36, 46). Follow-up time is concentrated on 6–12 months. The incidence of disabling spasticity increased over time. Meta-analysis showed overall an incidence of disabling spasticity and severe spasticity was 9.4% (95% CI 0.056 to 0.133, *I*^2^ = 87.8%; Figure 4); 2.5% (95% CI 0.009 to 0.041, *I*^2^ = 0.0%) in 1 month, 5% (95% CI 0.017–0.083) in 1–3 months, 12% (95% CI 0.084–0.157, *I*^2^ = 46.9%) in 6 months, 14.9% (95% CI 0.096–0.203, *I*^2^ = 46.5%) in 12 months. The incidence of severe spasticity was 10.3% (95% CI 0.058–0.149, *I*^2^ = 89.9%).

### Risk Factors for Spasticity

This meta-analysis included 15 cohort studies that reported one or more risk factors for PSS (24, 27, 30, 34, 36, 38, 39, 42, 43, 46, 48–51, 54). Two studies that did not use MAS or AS to assess spasticity were excluded from the meta-analysis of risk factors for PSS (31, 45). This meta-analysis found that BI, age, gender, hemisphere injury, smoking, hypertension, and diabetes could not predict PSS (Table 2). The heterogeneity among studies may be related to different study environment, subjects, evaluation methods, and time.

**Table 2:** Potential risk factors for spasticity after stroke in meta-analysis.

**Risk factors (subgroup)**	**Studies**	**Sample size**	**Effect**			
			**OR**	**95%CI**	***I*^**2**^**	***P***
Motor dysfunction	6	1,015	1.019	0.919–1.129	93.5%	0.723
Paresis	3	336	1.565	0.173–14.154	91.8%	0.690
Moderate to severe paresis			6.573	2.579–16.755	0%	*P* < 0.001
Sensory disorder	6	755	1.44	0.81–2.55	70.4%	0.217
1–3 months			1.226	0.493–3.052	34.5%	0.661
3–6 months			1.990	1.031–3.841		0.040
Over 6 months			0.850	0.725–0.997		0.045
Hemorrhagic stroke	4	3,504	1.879	1.418–2.490	27.3%	0.001
1 month	1	49	6.667	1.309–33.943	18.4%	0.022
3 months	2	3,128	1.469	0.933–2.312		0.097
6 months	1	3,056	1.680	1.235–2.285		0.001
12 months	1	3,056	1.844	1.375–2.472		*p* < 0.001
Barthel index	4	761	1.052	0.974–1.136	69.8%	0.195
Hypertension	4	723	1.431	0.939–2.181	23.1%	0.232
Age	6	679	0.963	0.885–1.048	73.4%	0.386
Gender	7	3,776	1.068	0.855–1.334	0.0%	0.561
Hemisphere injury	3	3,316	1.212	0.990–1.484	3.6%	0.062
Smoking	4	580	0.934	0.396–2.201	69.3%	0.876
Diabetes	3	478	1.174	0.890–1.405	44.2%	0.617
Posterior circulation	3	262	0.706	0.304–1.641	28.9%	0.418

*OR, odds ratio; CI, confidence interval*.

### Motor Dysfunction

Six studies with complete data explored whether motor dysfunction after stroke was a risk factor for PSS and enrolled one thousand seventeen participants (30, 36, 42, 43, 46, 48). Most studies suggested that motor dysfunction, especially early severe paresis, predicted PSS. Stroke patients with motor dysfunction was found to have no increased risk for PSS (OR = 1.019, 95% CI 0.919–1.129, *I*^2^ = 93.5, *P* = 0.723; Table 2). Subgroup analysis confirmed that there was no significant increase in the incidence of PSS in stroke patients with motor dysfunction throughout the stroke duration. A subgroup analysis was made on the possibility of predicting the occurrence of PSS with mild paresis and moderate to severe paresis. Mild paresis was defined as NIHSS score of less than or equal to 1 in item 5 or 6, or at grade 4 of the British Medical Research Council (BMRC) scale, while moderate to severe paresis was greater than or equal to 2 in NIHSS or BMRC grades 0–3. A subgroup of four studies that showed that only moderate to severe paresis was a risk factor for PSS (OR = 6.573, 95% CI 2.579–16.755, *I*^2^ = 0.0%, *P* < 0.001).

### Sensory Disorder

Six studies reported the effects of sensory disorders on PSS, involving 755 participants (30, 34, 42, 46, 50, 54). 2/3 studies concluded that sensory disorder was not associated with an increased risk of PSS, while another third found an association. The combined data showed no significant increase in the prevalence of PSS in patients with sensory disorder (OR = 1.44, 95% CI 0.81–2.55, *I*^2^ = 70.4%, *P* = 0.217; Table 2). In subgroup analysis, sensory disorder was found to predict PSS in 3–6 months (OR = 1.99, 95% CI 1.03–3.84, *I*^2^ = 34.5%; Table 2), but not at other times.

### Hemorrhagic Stroke

Five cohort studies compared the frequency of PSS in patients with ischemic or hemorrhagic stroke (30, 31, 34, 48, 49). A study was excluded from meta-analysis because of the use of diagnostic codes to assess PSS (31). Katoozian found a 2.5 times risk of PSS in patients with hemorrhagic stroke than ischemic stroke in 3 months (34). Lundström followed up 48 stroke patients and reported that 62.5% (5 of 8) of patients with hemorrhagic stroke and 20% with ischemic stroke developed PSS in 1 month (30). Pooled data suggested a significantly increased incidence of PSS in patients with hemorrhagic stroke (OR = 1.879, 95% CI 1.418–2.490, *I*^2^ = 27.3%, *P* < 0.001; Table 2). The risk of PSS was significantly increased at 1, 6, and 12 months in hemorrhagic stroke, but not at 3 months (OR = 1.469, 95% CI 0.933–2.312, *I*^2^ = 18.4%, *P* = 0.097; Table 2) (30, 34, 49).

### Stroke Site

Three studies with sufficient data provided an association between posterior circulation injury and poststroke spasticity (34, 42, 54). Combined results showed that posterior circulation injury was not the risk factor for PSS (OR = 0.706, 95% CI 0.304–1.641, *I*^2^ = 28.9%, *P* = 0.418). Because of insufficient data, fewer studies, and the classification of different stroke sites, we were unable to quantitatively update the relationship between other injury stroke sites and PSS. Some studies explored whether involvement of different stroke sites was a risk factor for PSS, especially for the upper limb spasticity. Jin found the highest incidence of upper limb spasticity (63.3%) in basal ganglia and internal capsule infarction (33). A retrospective study by Picelli revealed that damage to insula, thalamus, basal ganglia and white matter (internal capsule, corona radialis, external capsule and superior longitudinal tract) was significantly associated with severe PSS in upper limb (44). It was consistent as Cheung's finding (26). By using voxel-based lesion symptom mapping, Lee determined that corona radiata, posterior limb of internal capsule, thalamus, and putamen, premotor area, and insula were associated PSS in upper limb. Corona radiata, posterior limb of internal capsule, thalamus, and putamen caudate nucleus and external capsule are related to PSS in lower limb (40). In addition, the volume of stroke sites may also be associated with PSS. Ri found a high correlation between PSS and lesions in the middle cerebral artery supply area, pyramidal tract and/or internal capsule involvement and infarct size ≥3 cm^3^ (47). Moreover, extensive multiple infarction may be a risk factor for PSS (25, 54).

## Discussion

This systematic review and meta-analysis provide the first quantitative summary of most up to date prevalence of PSS and reviews the risk factors for PSS. PSS occurred in 25.3% of stroke patients and 39.5% of stroke patients with paresis, which was basically consistent with a previous review (55). Descriptive summary data from 24 studies showed a higher prevalence of PSS within 1 month (4–46%) than Wissel (4–27%) (3). This meta-analysis concluded that 9.4% of stroke patients with paresis developed severe or disabling spasticity, higher than the previous review of severe PSS in lower limb (below 6%) (15). Lower incidence may be associated with short follow-up time. The prevalence of disabling spasticity continued to increase within 12 months, in line with Sunnerhagen (56). Only one cohort study provided data on disabling spasticity, so more high-quality, long-term studies on disabling spasticity after stroke are needed in the future. The incidence of severe spasticity (10.3%) in stroke patients with paralysis was slightly higher than that of disabling or severe spasticity, suggesting that not all severe spasticity develops into disabling spasticity, consistent with Kong's result (35).

Two studies included patients with general stroke reported the incidence of severe spasticity (range from 2 to 2.6%). The paucity of studies on severe spasticity in the general stroke population are possibly due to the lower incidence of severe spasticity. General stroke patients are less likely to develop severe or disabling spasticity, and the prevalence of stroke patients with paresis is about 5 times as high as that of general stroke patients. Given the impacts of severe or disabling spasticity, stroke patients with paresis should be given more attention, especially with regard to the ability of activities or participation and quality of life.

This meta-analysis showed that 39.5% of stroke patients with paresis may have PSS, but the treatment needs of patients with PSS are different (16). Spasticity following a moderate to severe stroke tends to stabilize within 30–90 days after stroke with poorer function over time, and these patients require active attention and intervention. Patients with disabling spasticity must be distinguished from those with mild spasticity. Even the treatment of a minor disability may have similar economic benefits as the treatment of a more severe disability (57). It is necessary to use the ICF system or other reasonable methods to classify and manage patients with PSS. There are different mechanisms for motor recovery and spasticity (58, 59). The key to promoting motor recovery after stroke is to promote and regulate neuroplasticity through rehabilitation. By causing synaptic plasticity reorganization, Botulinum toxin creates a transient state of plasticity in the neuromotor system, allowing movement to be relearned and restored in the chronic phase (21, 60). However, the efficacy of botulinum toxin in combination with rehabilitation has not been well-documented (61).

The prevalence of PSS provides evidence for whether PSS occurs, but debates about when PSS occurs continues. Brunnstrom's theory characterized the onset of spasticity as a motor recovery phase and reflected PSS through synergistic effects rather than using AS or MAS to directly evaluate PSS. Onset of PSS helps to judge the prognosis of stroke patients and guide clinical treatment (62). A review by Sunnerhagen concluded that about half of patients with spasticity developed spasticity within 3 days after stroke, while the other half within the first month (56). Nam conducted a retrospective study of 861 stroke patients and found that median time from onset to onset of PSS in upper limb was 34 days. About half of patients developed PSS within 1 month after stroke, and a quarter of those with spasticity showed PSS after 2 months (63). However, Balakrishnan hold the opinion that PSS usually occurs within 1–6 weeks after stroke (64). Overall, the onset time of PSS is from 3 days to 6 weeks after stroke, mostly within 1 month. Changes in neuronal plasticity after central nervous system injury may lead to high variability in the onset of PSS (65). Neurogenic spasticity (spasticity and baseline activation) peaked 1–3 months after stroke (66). In the more advanced motor recovery stage, especially 3 months after stroke, resistance to passive movement was considered to be the result of inappropriate muscle activation (neural component) interacting with collagen tissue, tendons and muscle changes (biomechanical component) (12). As a widely used tool for evaluating PSS, MAS only provides subjective data on passive resistance and cannot distinguish the components of spasticity. And its poor reliability (67) and sensitivity may result in most patients being classified as having a moderate degree of clustering effect (68). The differentiation and objective quantification of spasmodic components should be combined with electrophysiological, biomechanical or optical evaluation methods to assist in clinical decision-making (69, 70).

Changes in PSS status help determine the follow-up time. This meta-analysis suggested that PSS usually appeared or disappeared in 1–3 months after stroke and remained stable beyond 3 months according to changes in the prevalence of PSS over time. Most patients change from spasticity to non-spasticity because the incidence of PSS was lower at 3 months than at 1 month. Different degrees of spasticity evolved differently over time. At three months after stroke, patients with mild spasticity were less likely to deteriorate over time, while nearly half of those with moderate spasticity progressed to severe spasticity, and those with severe spasticity remained stable (36). In addition, Opheim reported that changes in PSS mostly occurred from day 3 to day 10 and from day 10 to 4 weeks (41). However, changes of PSS within 1 month were not able to further explored because of the large variation in evaluation time. Future studies need to focus on the changes of PSS in early stroke.

Moderate to severe paresis was a risk factor for PSS, which was more detailed than motor dysfunction reported by previous meta-analysis (23). That's being expected. Reticulospinal tract and/or vestibulospinal tract hyperexcitability may be the mechanism of classic spasticity (71). After stroke, reticulospinal tract were activated and projected downward to both sides. Usually only in the paraplegia side developed spasticity may be due to stronger lateralization pathway that results in a marked increase in motor neuron excitability (59). Moreover, according to Brunnstrom's theory, patients with mild motor dysfunction may themselves be in or prone to developing into advanced motor recovery stages, and thus have a spasticity that eventually subsides or never experience spasticity (62).

Present evidence indicates that the possible sites associated with PSS, including internal capsule, basal ganglia, thalamus, insula, etc. Some pathophysiological evidences have been established that damage to these sites predicts PSS. Basal ganglia injury increases involuntary muscle activation and cortical ridge marrow injury reduces voluntary muscle control, which may lead to involuntary muscle overactivity in central paralysis (72). This is in line with the new definition of spasticity (6). In addition, damage to insula may result in disorders of the vestibulospinal system, which leads to PSS (21). Considering the association of posterior limb lesions with severe motor impairment or isolated recovery of upper limb motion, the involvement of internal capsule was expected (73). In addition, corticospinal tract inhibits spinal reflexes.

Another risk factor for PSS is hemorrhagic stroke, but no mechanism has been shown that hemorrhagic stroke directly predicted PSS. The most common sites of injury in hemorrhagic stroke were putamen/globus pallidus (56%) and internal capsule (51%). As discussed above, basal ganglia adjacent to internal capsule and corticospinal tract plays a role in predicting PSS. It's worth noting that hemorrhagic stroke may not be an independent risk factor for PSS, and damage to the basal ganglia region may lead to more PSS in patients with hemorrhagic stroke.

This study could not be specifically identified whether severe paresis, early increased muscle tone or poor motor control as risk factors for PSS, which have been reported by previous studies. We proposed to define severe paresis as BMRC grades 0–3. And an ideal kind of study trying to resolve the relationship between severe paresis and PSS should involve all stroke patients and followed up for more than half a year. In addition, this review lacked data from the less developed region of Africa, where the lack of primary health-care services and increased the risk of disability. Emphasis should be placed on prevention and rehabilitation with post-stroke disability in these areas (74).

## Study Limitations

This meta-analysis could not identify risk factors for severe spasticity or disabling spasticity because of different assessment methods and fewer studies. Stroke sites could not be subjected to a meta-analysis due to insufficient data after contacting the authors. Due to few studies, meta-regression analysis could not be conducted to explore the great heterogeneity. Publication bias risk assessment could not be carried out for the same reason. Due to the lack of long-term follow-up studies, this review was unable to explore the incidence of disabling spasticity more than 1 year after stroke. Since only the risk factors reported in at least three studies were included in the meta-analysis, it was not possible to analyze the effects of early increased muscle tone, NHISS scores and some stroke sites on the prediction of PSS.

## Conclusions

Overall, the incidence of PSS was 25.3%. 39.5% patients after first-ever stroke with paresis showed spasticity and 9.4% of which developed into severe or disabling spasticity. For patients with moderate to severe paresis, sensory disorder, close follow-up and intervention should be strengthened. The role of hemorrhagic stroke in predicting PSS remains to be further explored. Disabling spasticity requires longer follow-up studies and early identification and intervention are necessary due to impairment of function.

## Data Availability Statement

The original contributions presented in the study are included in the article/Supplementary Material, further inquiries can be directed to the corresponding author/s.

## Author Contributions

HZ and JC have been involved at every step of the research process and have made similar contributions to the work. ST and YG have put forward the research program of this study and guided the whole process of this study. All authors contributed to the article and approved the submitted version.

## Conflict of Interest

The authors declare that the research was conducted in the absence of any commercial or financial relationships that could be construed as a potential conflict of interest.

## Supplementary Material

Supplementary Figure 1 Prevalence of PSS in first ever stroke patients.

Supplementary Figure 2 Prevalence of severe PSS in patients with paresis.

Supplementary Figure 3 Forest plot of the OR analysis for damaged hemisphere and poststroke spasticity.

Supplementary Figure 4 Forest plot of the OR analysis for diabetes and poststroke spasticity.

Supplementary Figure 5 Forest plot of the OR analysis for gender and poststroke spasticity.

Supplementary Figure 6 Forest plot of the OR analysis for hypertension and poststroke spasticity.

Supplementary Figure 7 Forest plot of the OR analysis for smoking and poststroke spasticity.

Supplementary Figure 8 Forest plot of the OR analysis for motor dysfunction and poststroke spasticity.

Supplementary Figure 9 Forest plot of the OR analysis for motor dysfunction and poststroke spasticity over time.

Supplementary Figure 10 Forest plot of the OR analysis for different degrees of paresis and poststroke spasticity.

Supplementary Figure 11 Forest plot of the OR analysis for sensory disorder and poststroke spasticity.

Supplementary Figure 12 Forest plot of the OR analysis for sensory disorder and poststroke spasticity over time.

Supplementary Figure 13 Forest plot of the OR analysis for hemorrhagic stroke and poststroke spasticity.

Supplementary Figure 14 Forest plot of the OR analysis for hemorrhagic stroke and poststroke spasticity over time.

Supplementary Figure 15 Forest plot of the OR analysis for posterior circulation and PSS.
