# Supplementary figures and images for: Prevalence and Risk Factors for Spasticity After Stroke: A Systematic Review and Meta-Analysis
Source: Front Neurol. 2021 Jan 20;11:616097. doi: 10.3389/fneur.2020.616097 (PMC7855612; doi:10.3389/fneur.2020.616097)

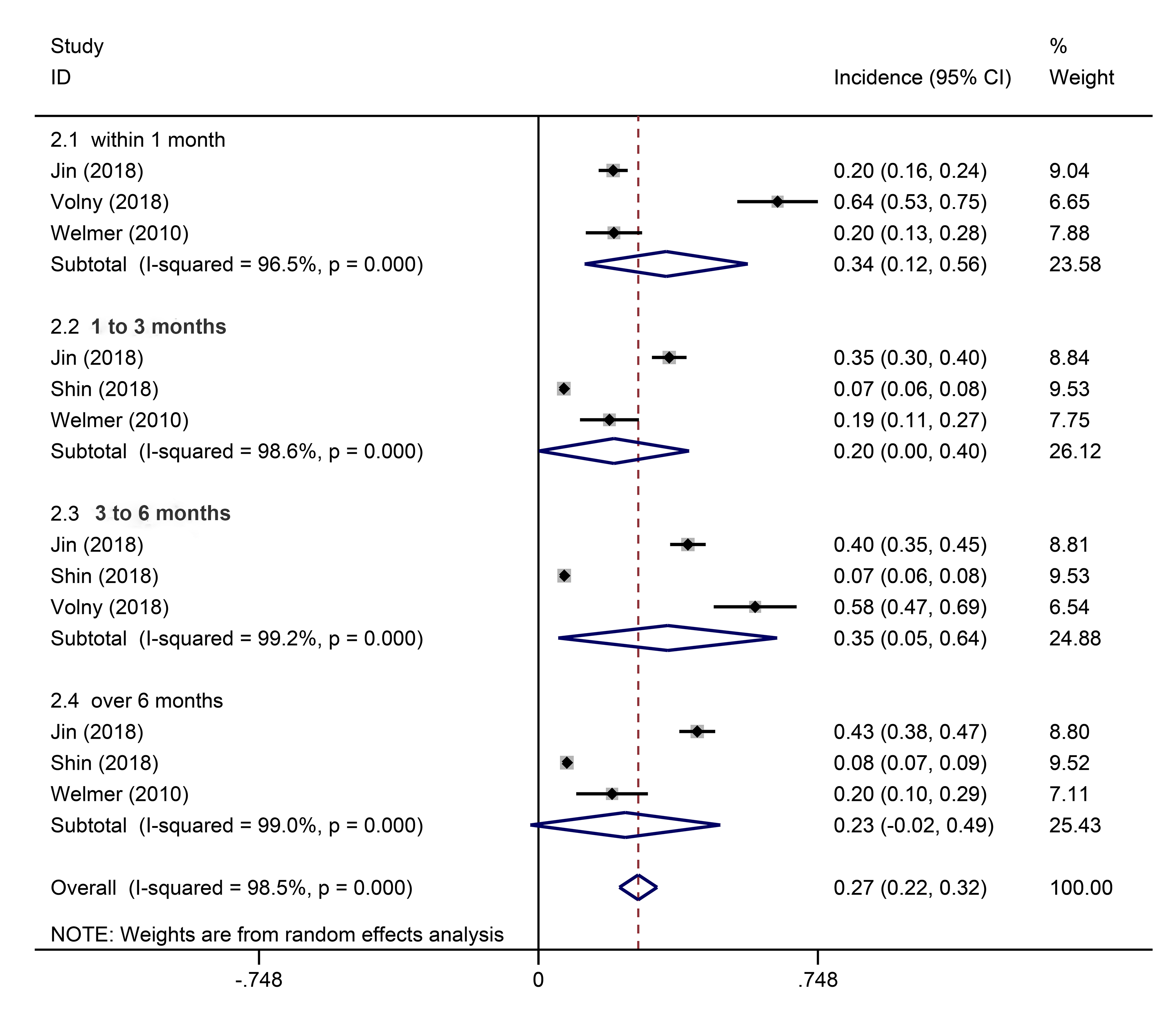

Supplement: Supplementary Figure 1 — Prevalence of PSS in first ever stroke patients. [file Image_1.TIF]

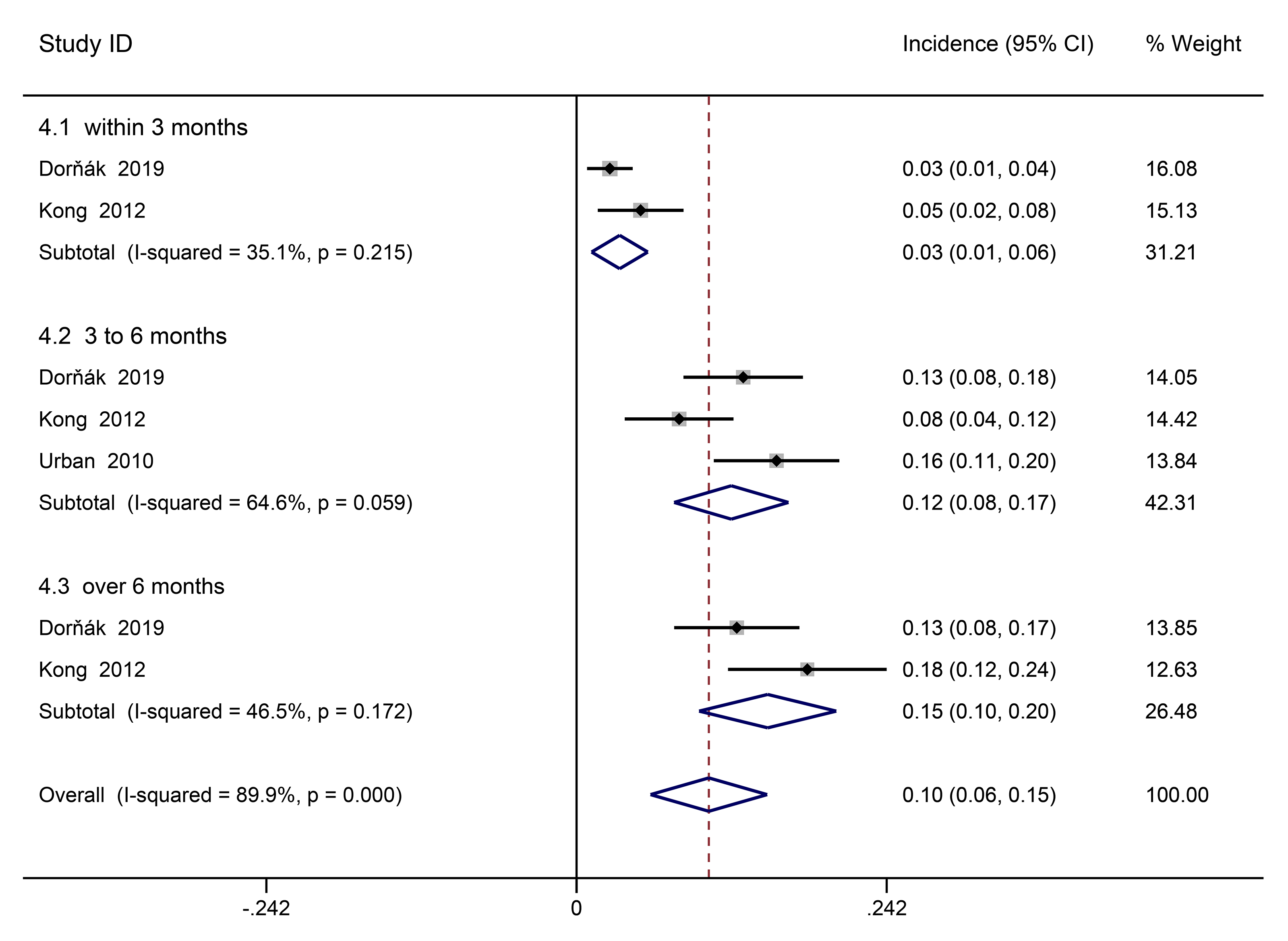

Supplement: Supplementary Figure 2 — Prevalence of severe PSS in patients with paresis. [file Image_2.TIF]

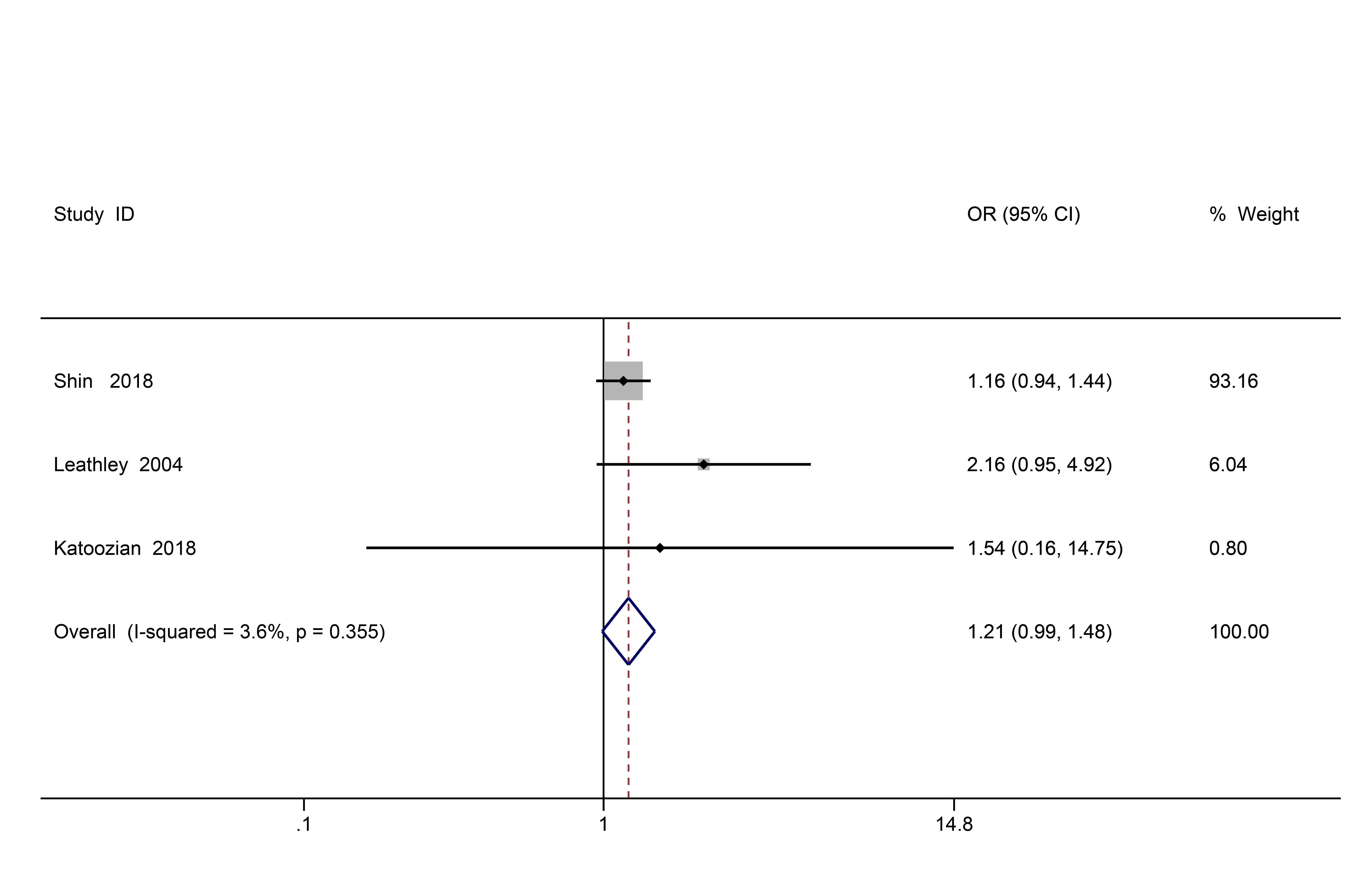

Supplement: Supplementary Figure 3 — Forest plot of the OR analysis for damaged hemisphere and poststroke spasticity. [file Image_3.TIF]

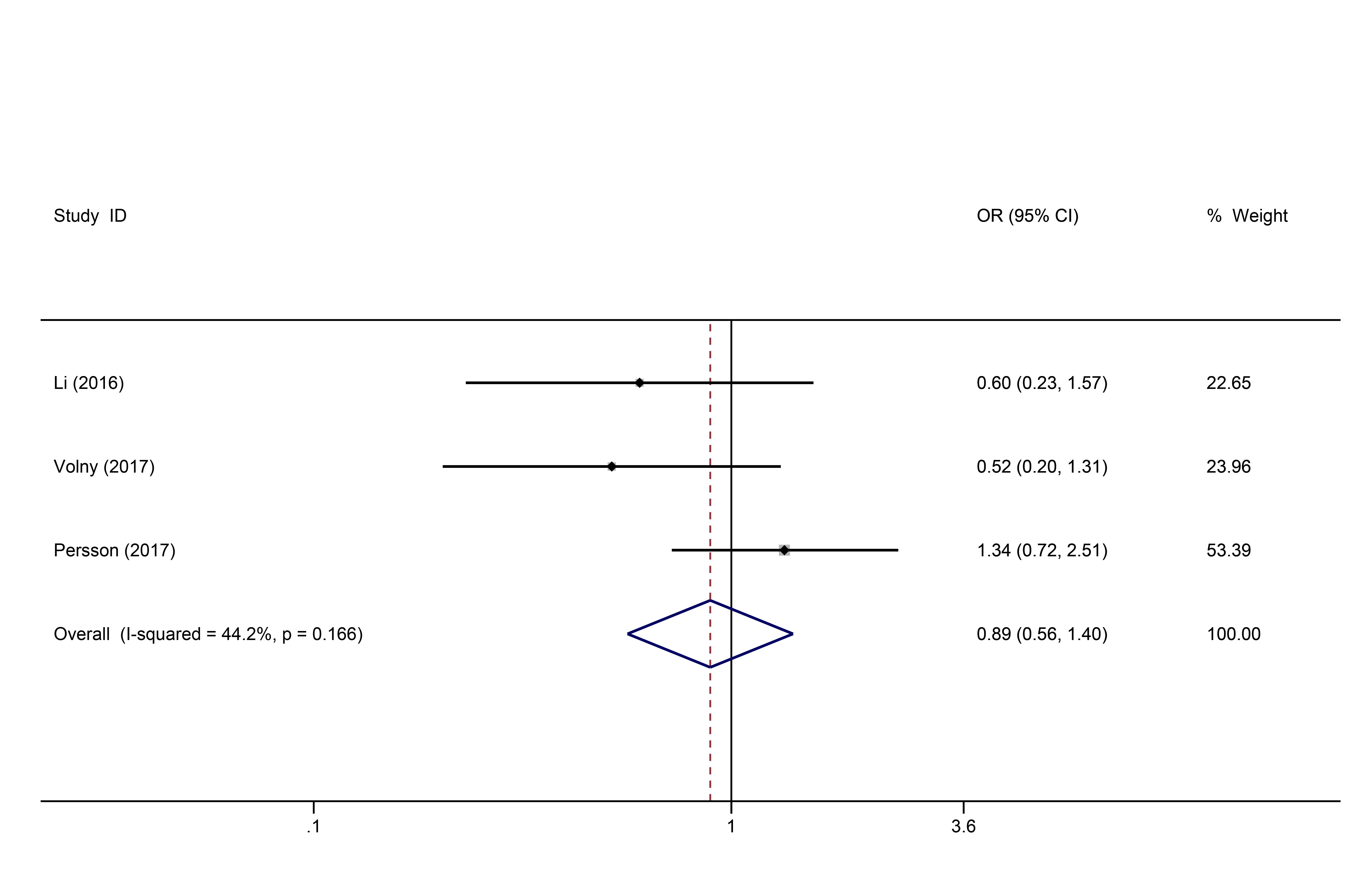

Supplement: Supplementary Figure 4 — Forest plot of the OR analysis for diabetes and poststroke spasticity. [file Image_4.TIF]

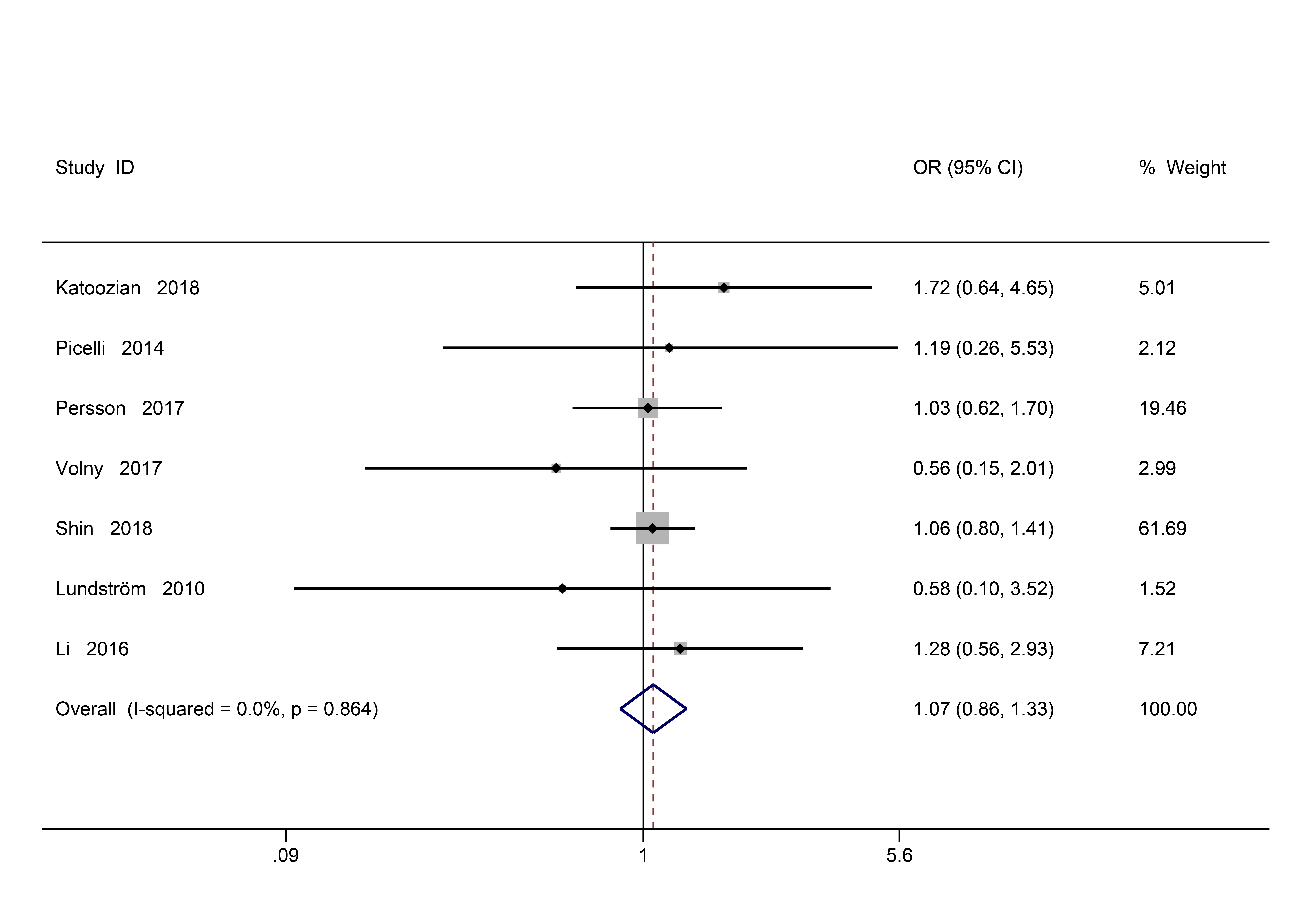

Supplement: Supplementary Figure 5 — Forest plot of the OR analysis for gender and poststroke spasticity. [file Image_5.TIF]

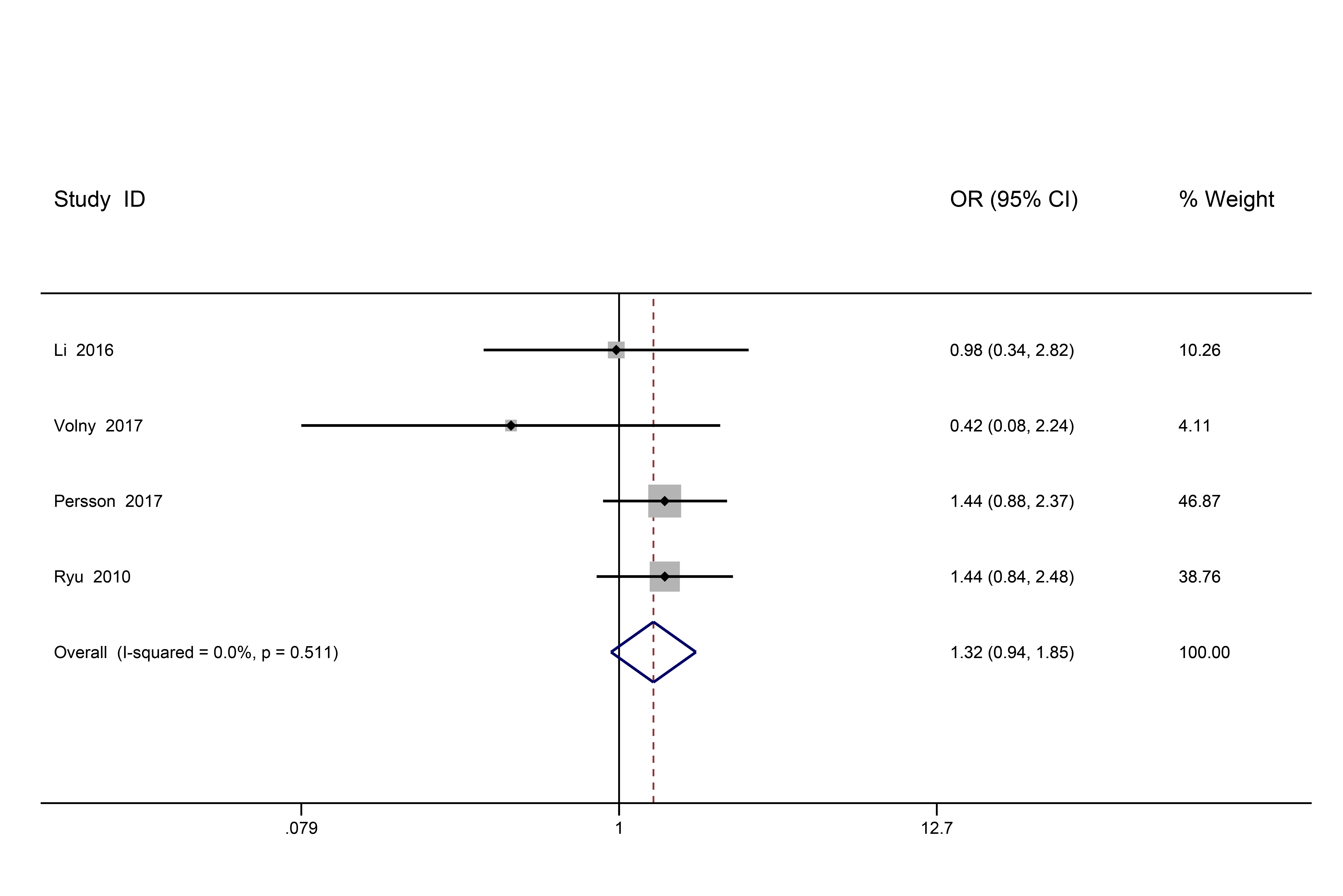

Supplement: Supplementary Figure 6 — Forest plot of the OR analysis for hypertension and poststroke spasticity. [file Image_6.TIF]

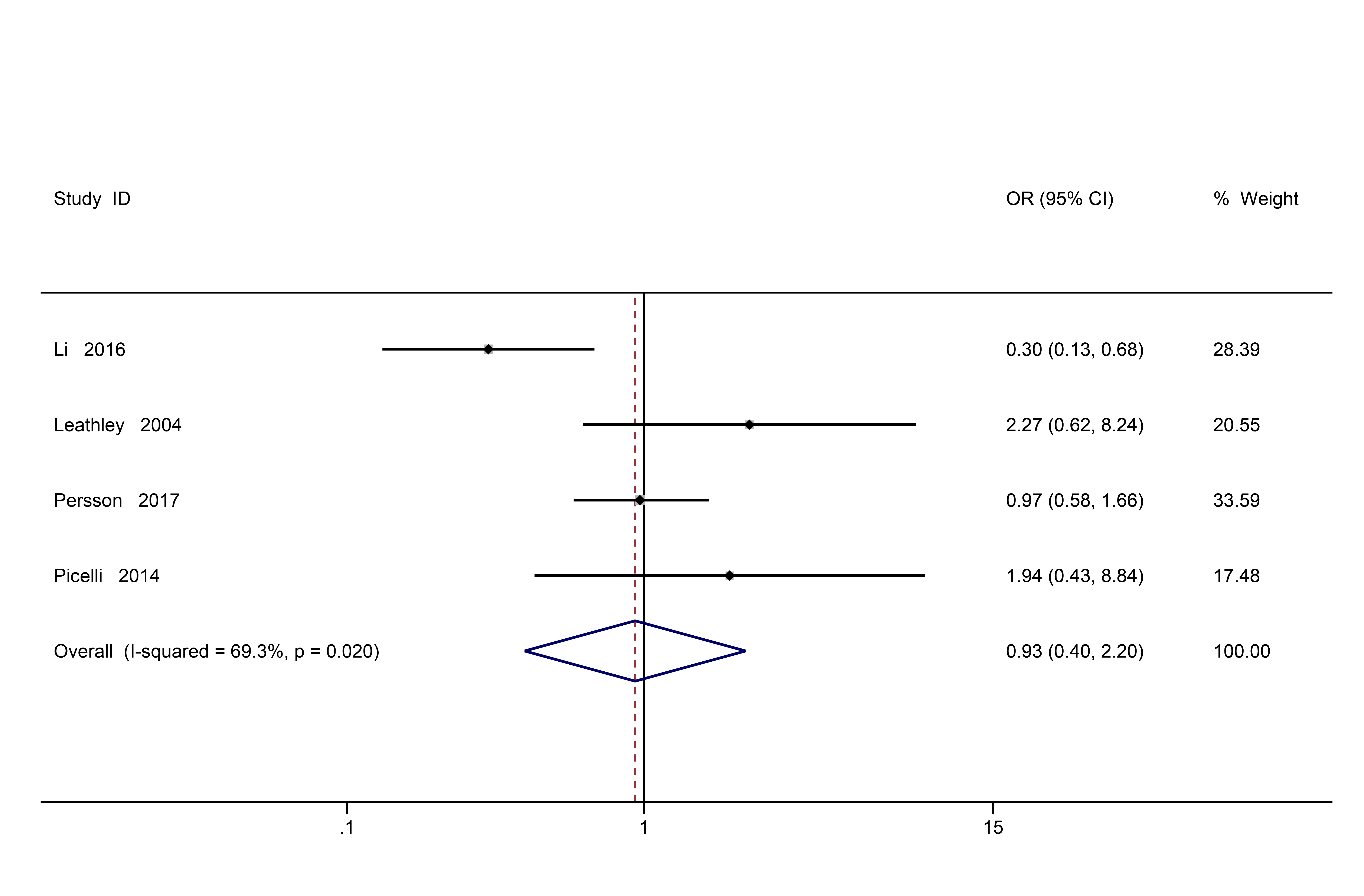

Supplement: Supplementary Figure 7 — Forest plot of the OR analysis for smoking and poststroke spasticity. [file Image_7.TIF]

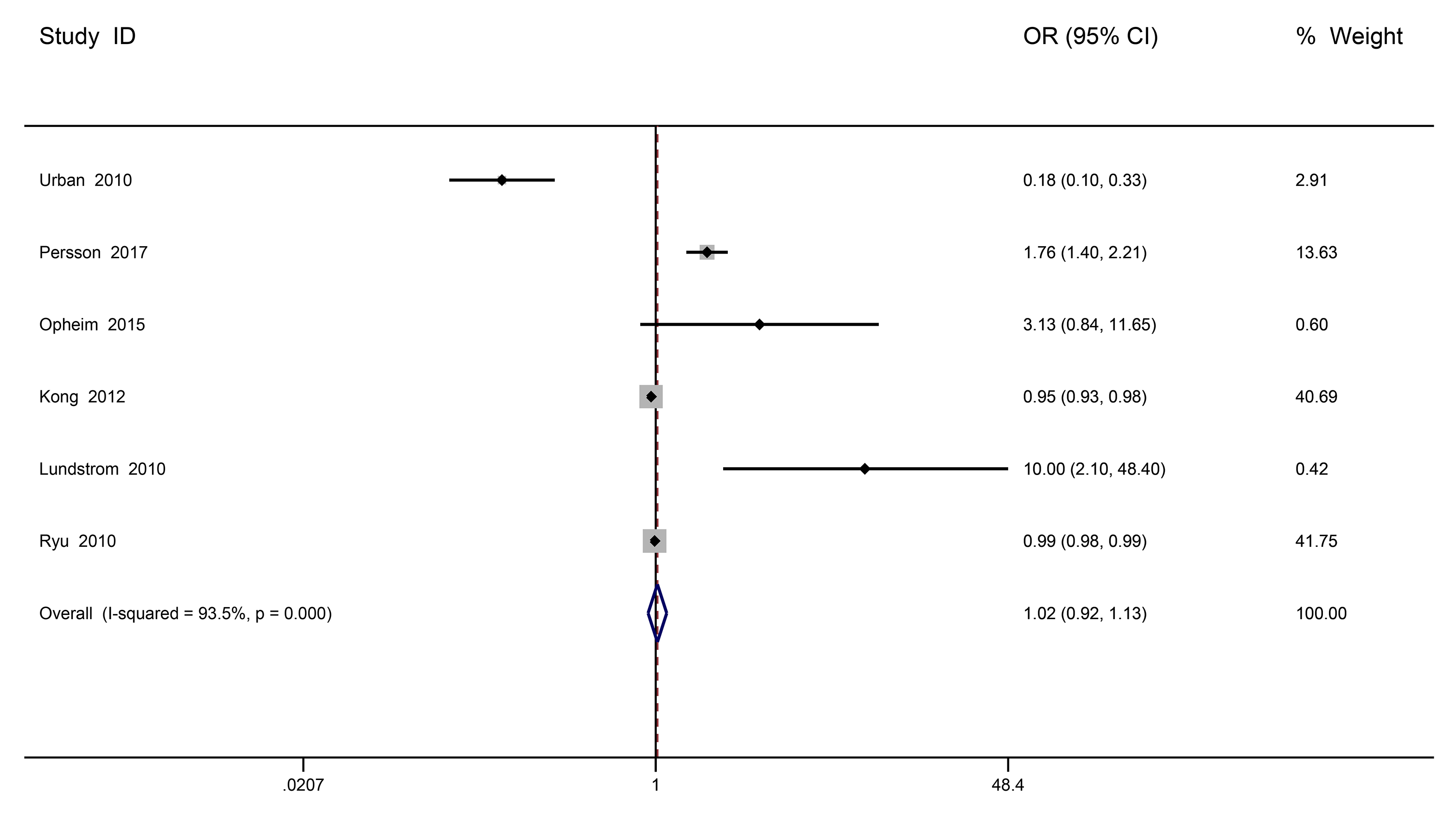

Supplement: Supplementary Figure 8 — Forest plot of the OR analysis for motor dysfunction and poststroke spasticity. [file Image_8.TIF]

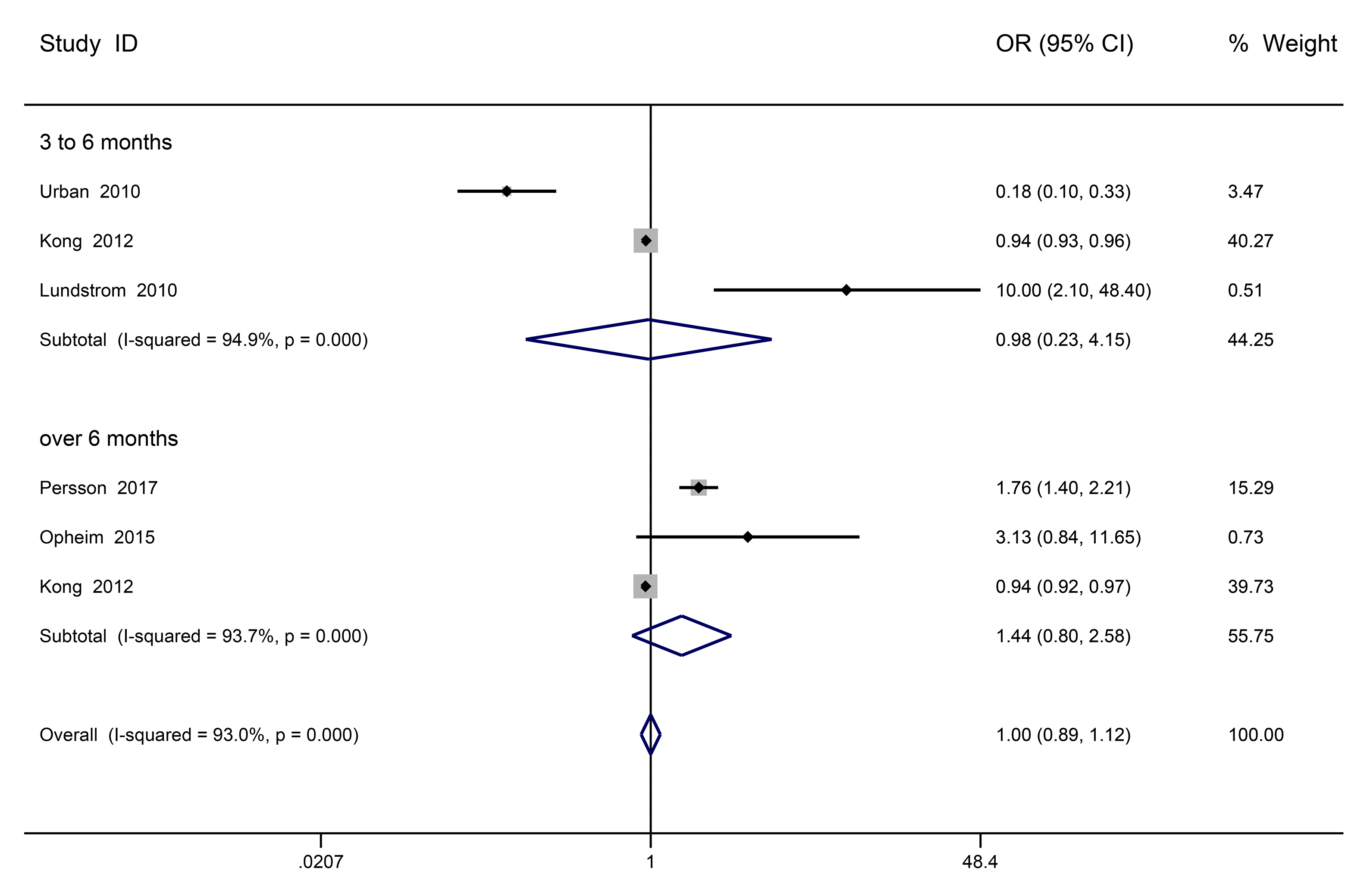

Supplement: Supplementary Figure 9 — Forest plot of the OR analysis for motor dysfunction and poststroke spasticity over time. [file Image_9.TIF]

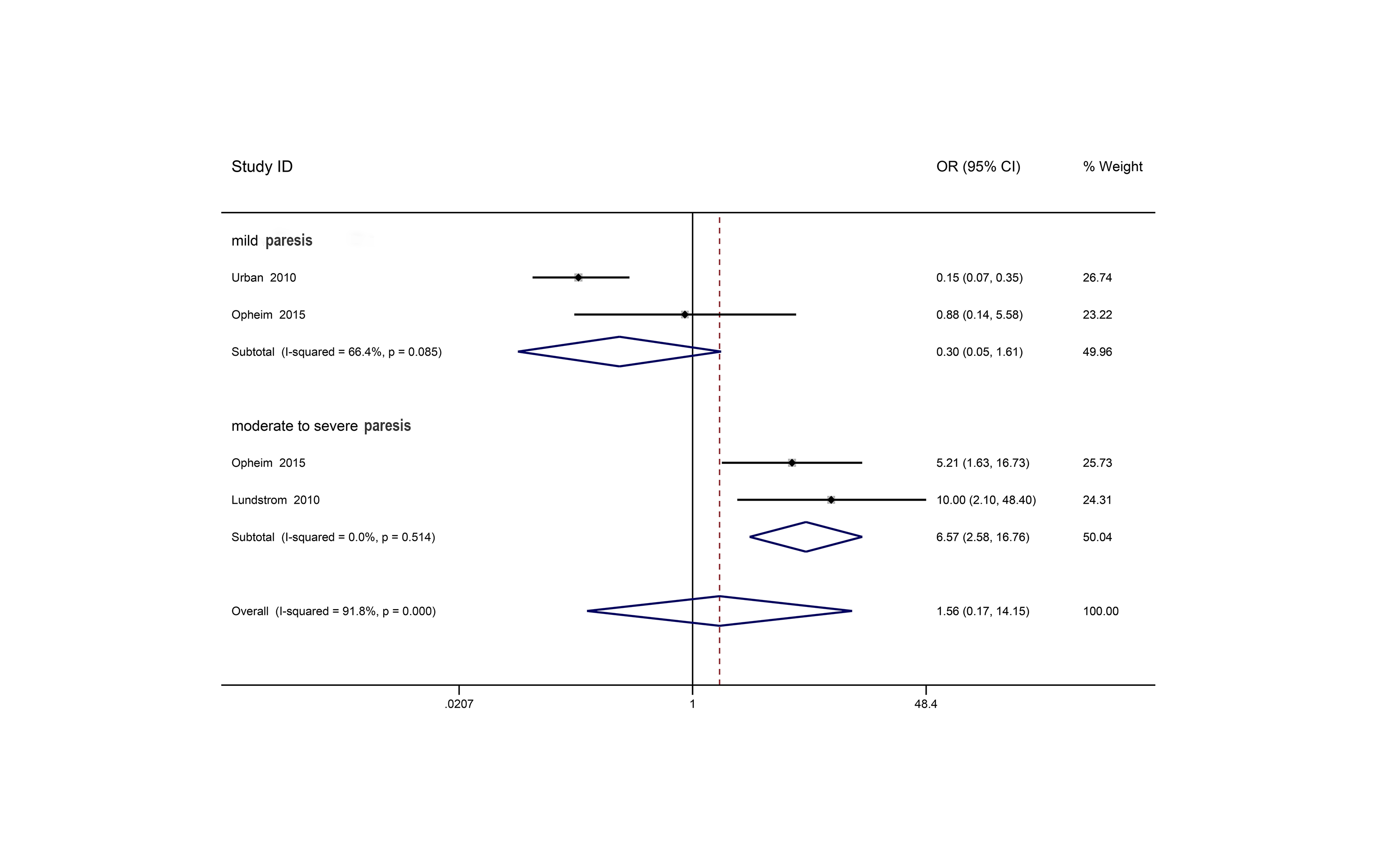

Supplement: Supplementary Figure 10 — Forest plot of the OR analysis for different degrees of paresis and poststroke spasticity. [file Image_10.tif]

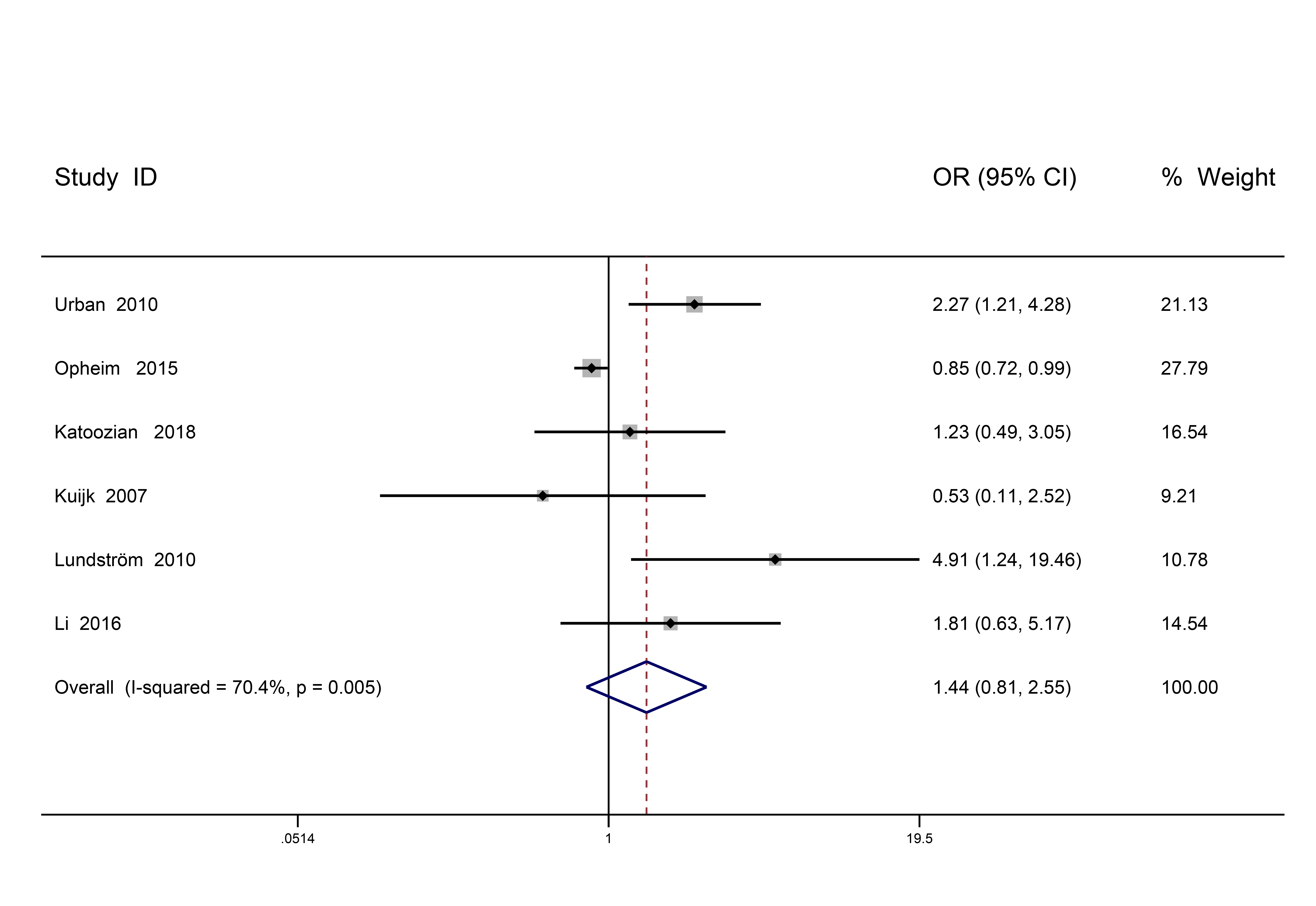

Supplement: Supplementary Figure 11 — Forest plot of the OR analysis for sensory disorder and poststroke spasticity. [file Image_11.TIF]

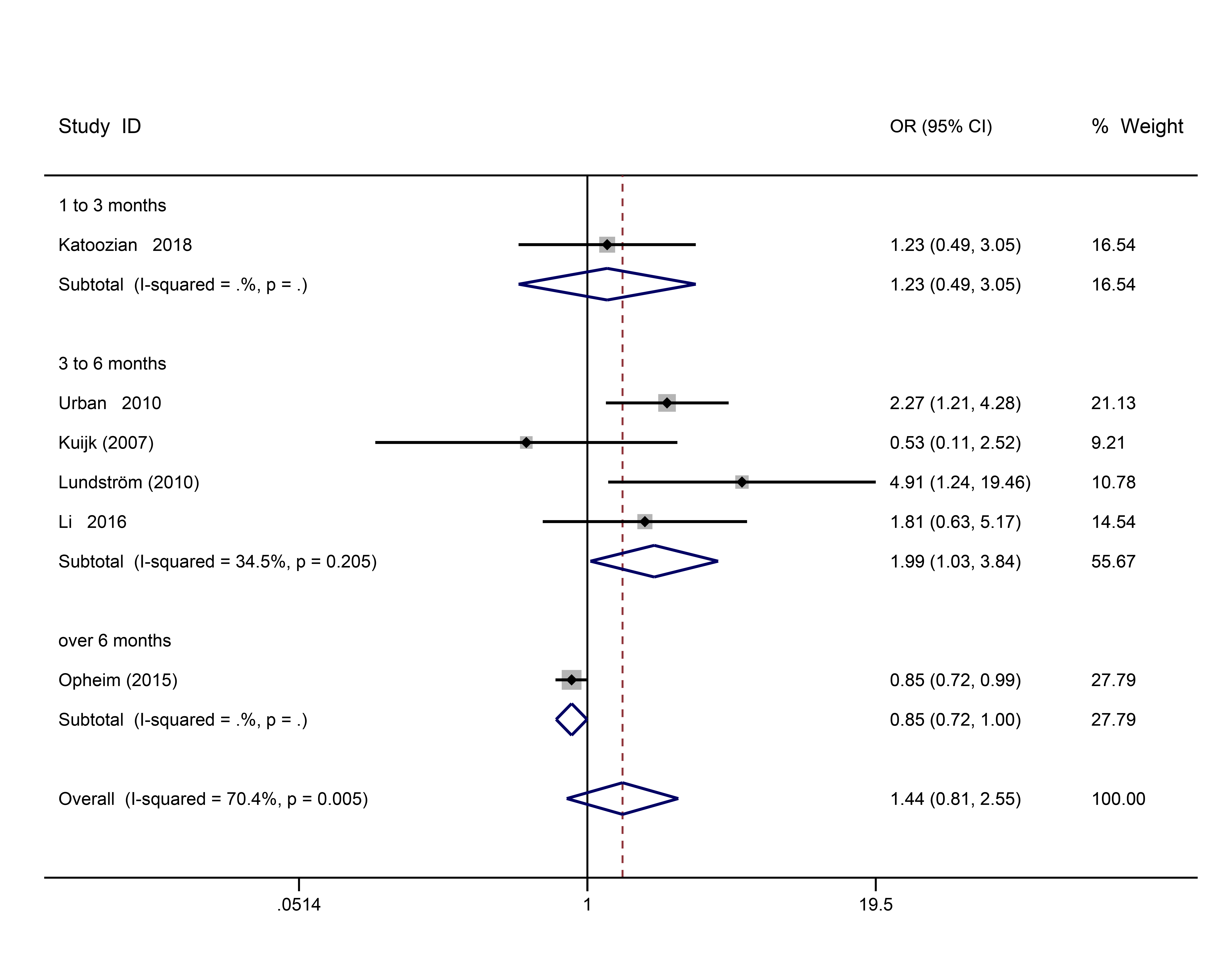

Supplement: Supplementary Figure 12 — Forest plot of the OR analysis for sensory disorder and poststroke spasticity over time. [file Image_12.TIF]

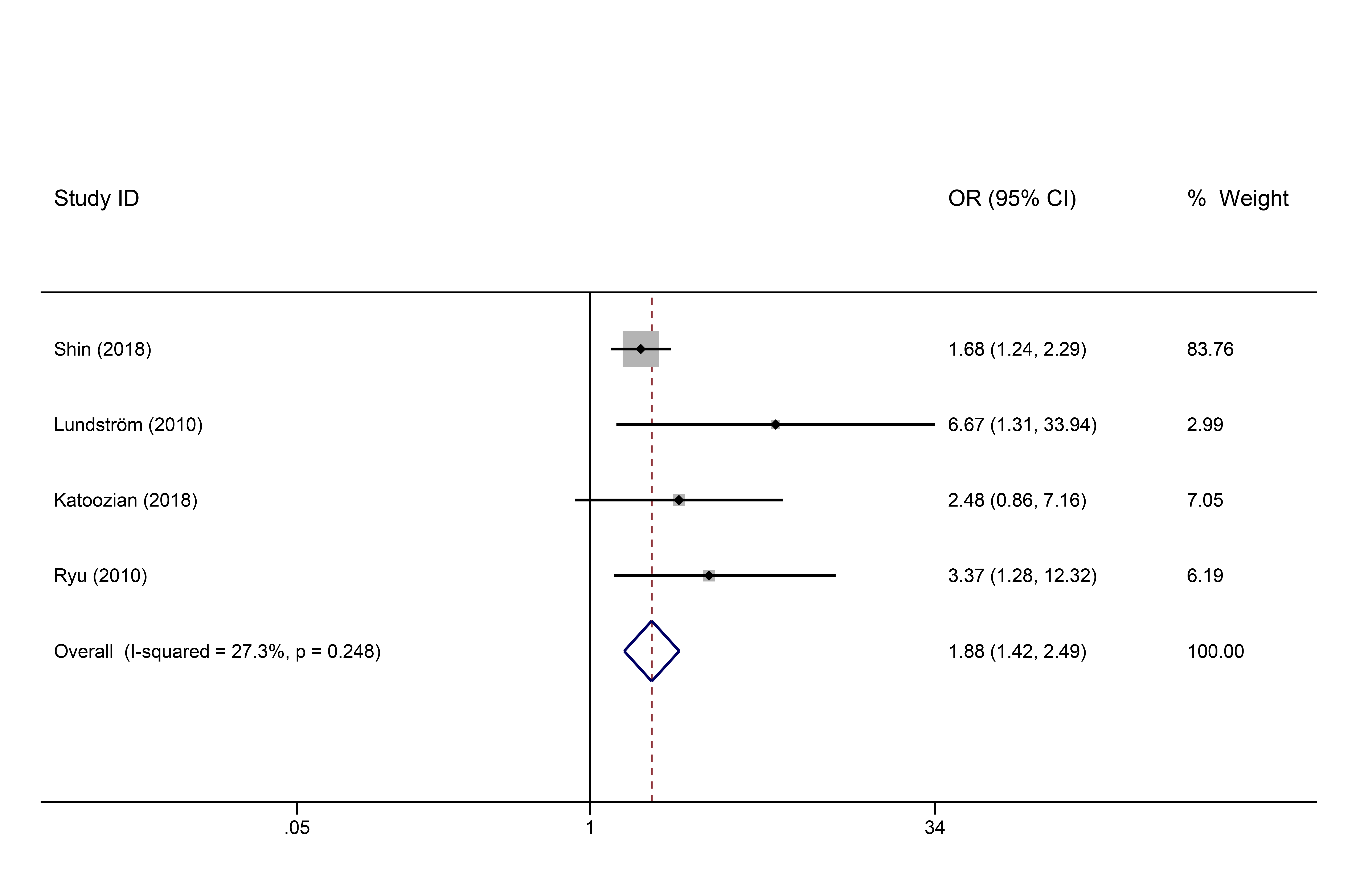

Supplement: Supplementary Figure 13 — Forest plot of the OR analysis for hemorrhagic stroke and poststroke spasticity. [file Image_13.TIF]

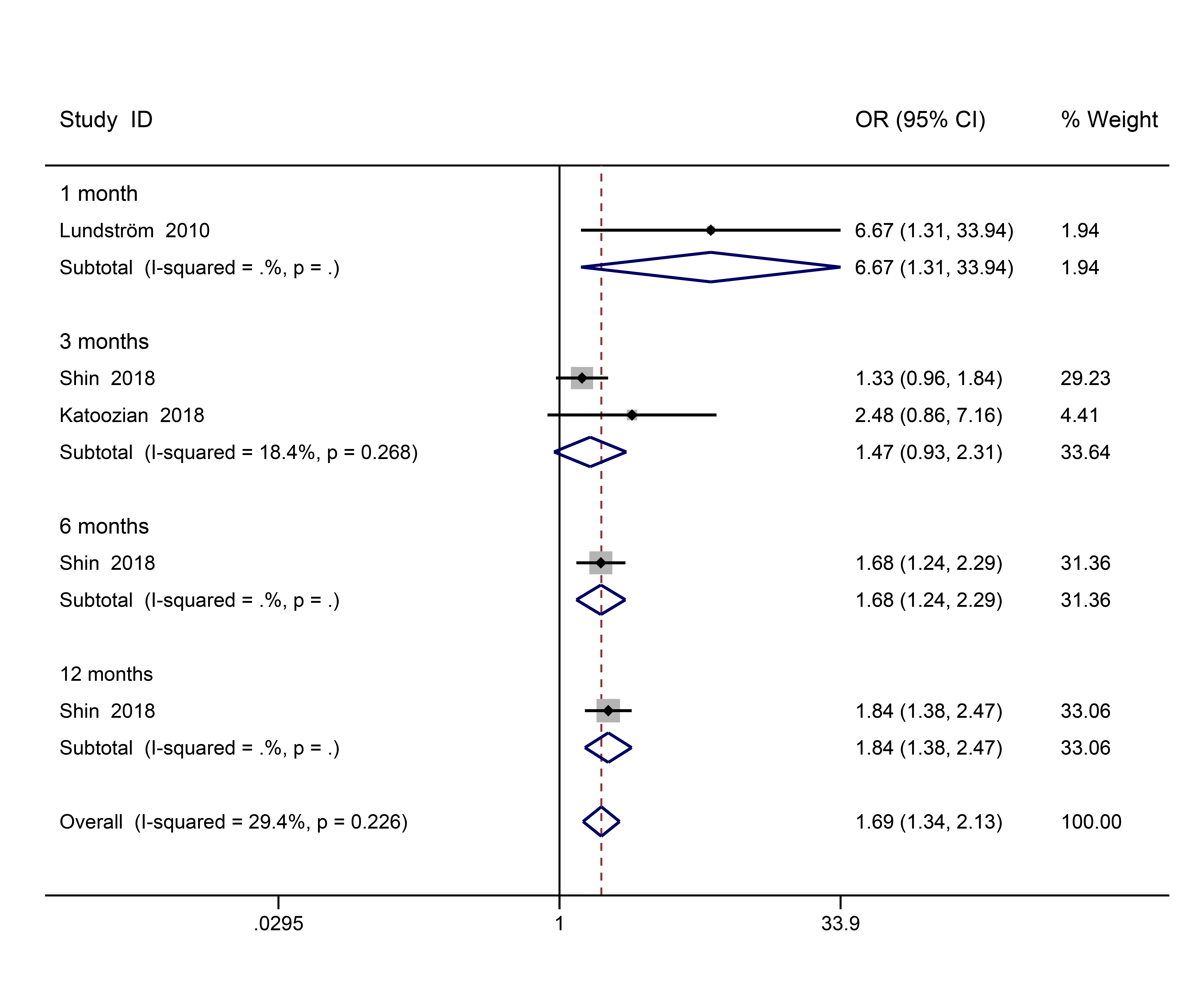

Supplement: Supplementary Figure 14 — Forest plot of the OR analysis for hemorrhagic stroke and poststroke spasticity over time. [file Image_14.TIF]

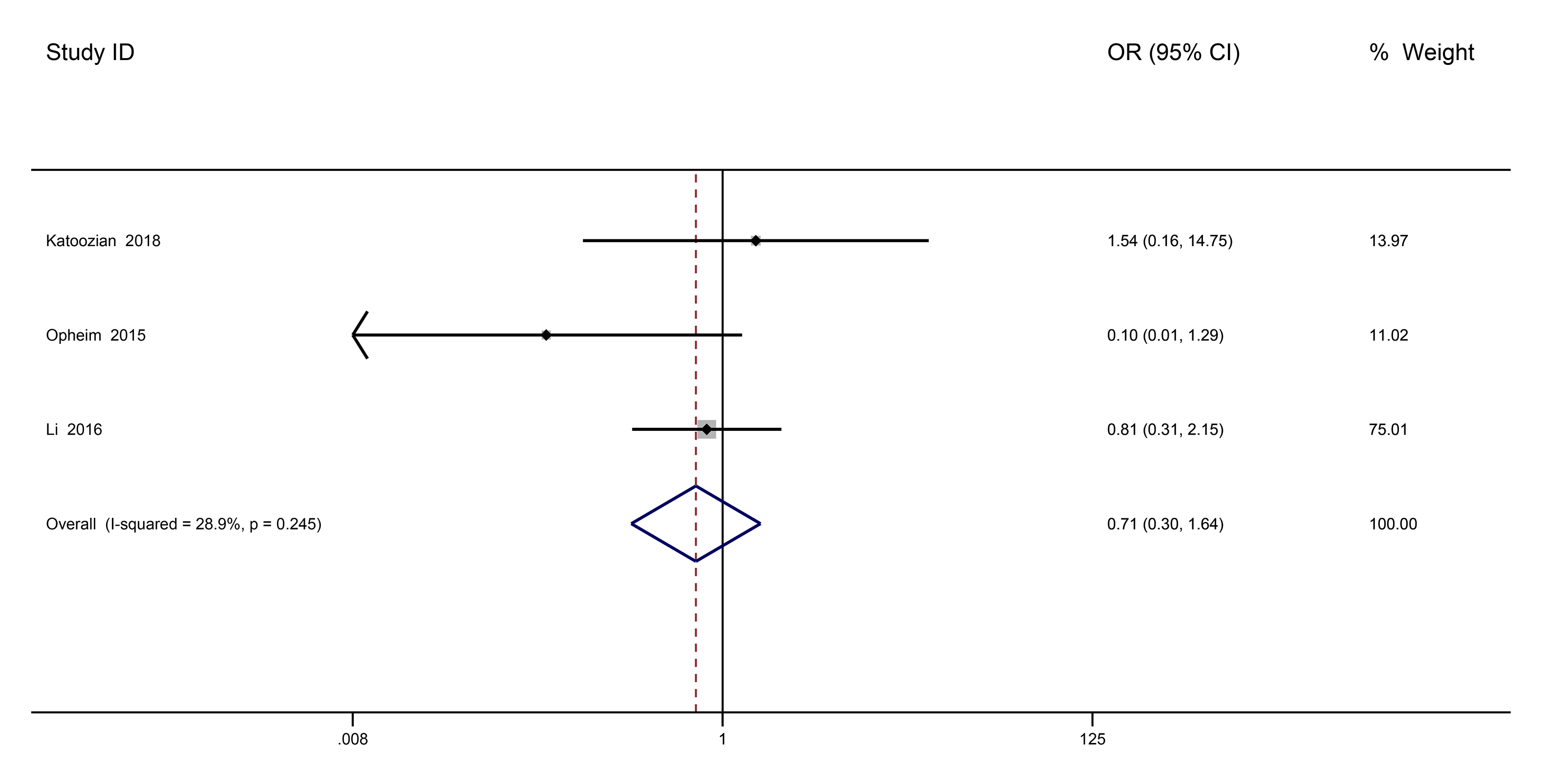

Supplement: Supplementary Figure 15 — Forest plot of the OR analysis for posterior circulation and PSS. [file Image_15.TIF]
